# Biocoordination reactions in copper(II) ions and phosphocholine systems including pyrimidine nucleosides and nucleotides

**DOI:** 10.1038/s41598-023-37986-1

**Published:** 2023-07-04

**Authors:** Malwina Gabryel-Skrodzka, Martyna Nowak, Jakub Grajewski, Renata Jastrząb

**Affiliations:** Faculty of Chemistry, Adam Mickiewicz University, 61–614 Poznan, Poland

**Keywords:** Bioinorganic chemistry, Metals, Analytical chemistry, Coordination chemistry, Inorganic chemistry

## Abstract

The complexation reactions of phosphocholine and pyrimidine nucleosides as well as nucleotides with copper(II) ions were studied in the water system. Using potentiometric methods and computer calculations, the stability constants of the species were determined. Using spectroscopic methods such as UV-vis, EPR, ^13^C NMR, ^31^P NMR, FT–IR and CD, the coordination mode was established for complexes created in pH range 2.5–11.0. These studies will lead to a better understanding the role of copper(II) ions in living organisms and explain the interactions between them and the studied bioligands. The differences and similarities between nucleosides and nucleotides in the studied systems were also described, which testify to the significant influence of phosphate groups on the processes of metal ion complexation and interactions between ligands.

## Introduction

**Figure 1:**
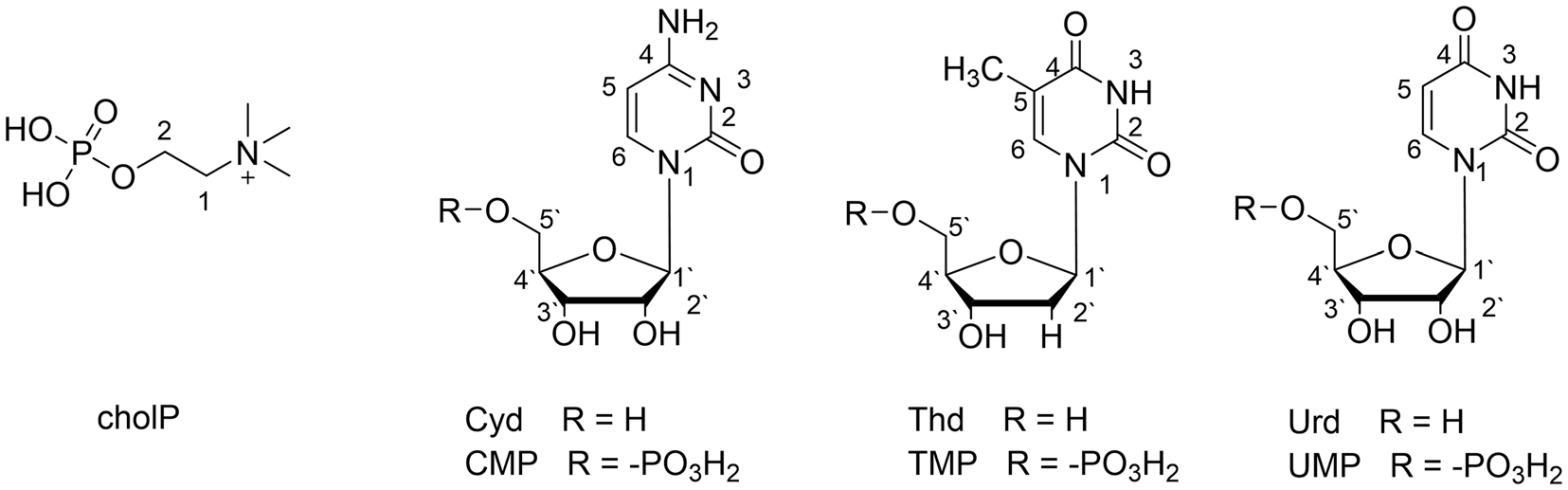
Chemical formula of the studied bioligands.

Phosphocholine (cholP) (Fig. 1) is a hydrophilic part of phosphatidylcholine (PC)—one of the most abundant phospholipid^1^. PC plays an important role in the absorption and transport of dietary fat^2^. Phosphatidylcholines are a major component of biological membranes. Lecitines are involved in various metabolic processes, are very important components of the brain and nervous tissue—they protect the myelin sheath, form a protective barrier for the walls of the stomach, and participate in cholesterol management. For these reasons, they are studied for structural, mechanical and electrical properties^3–6^. The biological membranes they form are exposed to different pH values of the environment in living organisms. Changes in the pH value of the environment affect the stability of biomembranes: zwitter–ionic lipid molecules contain functional groups that can interact with hydrogen or hydroxide ions, changing the charge density of biomembranes^7^. Also, the pH value affects the transport across lipid bilayers.

Phosphatidylcholine in living organisms can be synthesized in several ways. One of them is the Kennedy pathway. Choline, supplied from food, including eggs, dairy products and meat, undergoes phosphorylation catalyzed by choline kinase. Subsequently, phosphocholine reacts with cytidine-5`-triphosphate in the presence of CTP:phosphocholine cytidylyltransferase and this process is the rate—determining step in the pathway. Subsequently, CDP–choline condenses with diacylglycerol to produce PC^8–10^.

Phosphocholine is one of the binding targets of C–reactive protein. CRP binds to cholP when the cell is damaged which initiate recognition and phagocytotic immune response^11,12^.

In addition, cholP could be used as a biomarker of breast cancer^13,14^. In general, the concentration of choline and its derivatives is stable and equals 5–10 µM. Higher concentrations of these metabolites have been correlated with human breast cancer cells^15^. Furthermore, an increase in cholP level in the cerebrospinal fluid was observed in cerebrospinal fluid in patients with Alzheimer`s disease^16–18^.

The properties of biological membranes are influenced not only by the pH of the environment, but also by the presence and amount of metal cations with which they can interact. One of them are copper ions, which, as cofactors of many proteins in living organisms, have important functions including in electron transfer, oxygen respiration, and antioxidant processes^19^.

This article presents the results of potentiometric and spectral studies of the complexation of phosphocholine and pyrimidine nucleosides as well as nucleotides with copper(II) ions. These studies can contribute significantly to a better understanding of the interaction of copper ions with the ligands under study over a wide range of pH, especially within the nervous system, where cholP–containing lipids are abundant. On the subject of complexes of nucleosides and nucleotides with copper (II) in binary and ternary systems, it is able to find quite a lot of information^20–22^. Ternary systems described in the literature, in addition to copper ions, nucleosides or nucleotides, contain e.g. amino acids, polyamines, peptides, imidazole and its derivatives or chitosan^23–26^. Phosphocholine ternary complexes have not been previously described.

## Results and discussion

The structures of the ligands covered by the research presented in this work are shown in the Fig. 1. On the basis of potentiometric titration data, the protonation constant, the overall and the successive stability and equilibrium constants were determined using the HYPERQUAD program^27^. The rightness of the choice of the type of complexes was confirmed by the results of computer analysis in which experimental and theoretical curves converged.

The protonation constants of the studied nucleosides and nucleotides as well as stability constants of the binary complexes of them were reported by our research group before (Table 1). For phosphocholine, these values were determined and presented in this paper. Subsequently, potentiometric and spectroscopic studies were performed for the ternary Cu(II)/cholP/Nuc and Cu(II)/cholP/NMP systems.

### Binary Cu(II)/cholP system

#### Potentiometric studies of the Cu(II)/cholP system

In the first step, the protonation constant of phosphocholine log*β* = 5.48 was determined by computer calculations from the titration data and is consistent with the data from the literature (Table 1)^16,28,29^. The first proton from the phosphate group dissociates below the investigated pH value, and the first protonation constant of the ligand was not determined.

**Table 1:** Protonation constants of the studied ligands and stability constants for complexes in Cu(II)/L systems.

Species	cholP	Cyd^22^	CMP^22^	Thd^22^	TMP^30^	Urd^22^	UMP^25^
H_2_L	–	–	10.90	–	15.77	–	15.13
HL	5.48(1)	4.49	6.42	9.79	9.73	9.22	9.50
MHL	–	–	–	–	13.57	11.55	–
ML_2_H	13.21(7)	–	–	–	–	–	–
ML	–	2.25	2.71	5.67	–	4.32	6.03
M_2_L_2_	–	–		–		12.86	–
ML(OH)	–	–	− 4.26	− 1.92	0.39	− 3.72	− 2.82
ML(OH)_2_	− 9.50(3)	− 12.09	–	− 9.76	− 9.24	− 12.96	− 13.02
ML(OH)_3_	–17.67(4)	–	–	− 16.48	–	− 23.78	− 23.64

Taking into account the designated value, potentiometric titrations were performed in a metal:ligand ratio of 1:1. The hydrolysis constant value for Cu(OH)_2_ used for the calculations was log*β* = −13.13^31^. The protonated form of the complex and hydroxocomplexes with their equilibrium constants were established on the proposed reaction of their formation:

$${\text{Cu}}^{{{2} + }} + \left( {{\text{HcholP}}} \right) + \left( {{\text{cholP}}} \right) \leftrightarrows {\text{CuH}}\left( {{\text{cholP}}} \right)_{{2}} \quad {\text{log}}K_{{\text{e}}} = {7}.{73}$$

$${\text{Cu}}^{{{2} + }} + \left( {{\text{cholP}}} \right) + {\text{2H}}_{{2}} {\text{O}} \leftrightarrows {\text{Cu}}\left( {{\text{cholP}}} \right)\left( {{\text{OH}}} \right)_{{2}} + {\text{2H}}^{ + } \quad {\text{log}}K_{{\text{e}}} = { 8}.0{4}$$

$${\text{Cu}}\left( {{\text{cholP}}} \right)\left( {{\text{OH}}} \right)_{{2}} + {\text{ H}}_{{2}} {\text{O}} \leftrightarrows {\text{Cu}}\left( {{\text{cholP}}} \right)\left( {{\text{OH}}} \right)_{{3}} + {\text{ H}}^{ + } \quad {\text{log}}K_{{\text{e}}} = { 5}.{6}0$$

**Figure 2:**
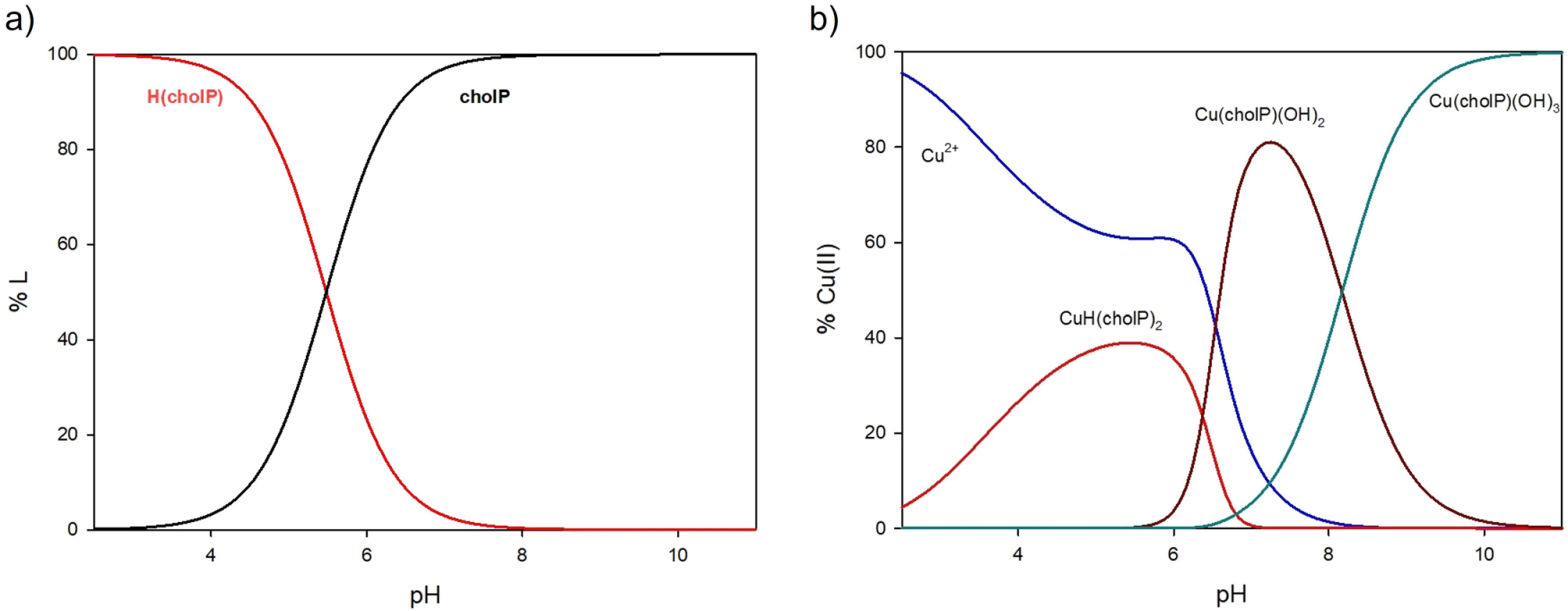
Distribution diagrams for (**a**) protonation of cholP (**b**) Cu(II)/cholP system.

At pH 2.5, Cu^2+^ ions and CuH(cholP)_2_ complex were observed. At first, the complex binds only 5% of total copper(II) ions (Fig. 2). At pH 5.5 it reaches its maximum concentration in solution binding about 40% of the metal ions. From pH 6.0 to 10.0 the first hydroxocomplex Cu(cholP)(OH)_2_ exists and it dominates at pH 7.2 binding more than 80% of Cu^2+^. As shown in Fig. 2, the formation of Cu(cholP)(OH)_3_ starts at pH = 6.0 and the maximum amount of Cu(II) complexing occurs at pH 10.0 (100% of the metal ions introduced into solution).

#### Spectroscopic studies of the Cu(II)/cholP system

**Figure 3:**
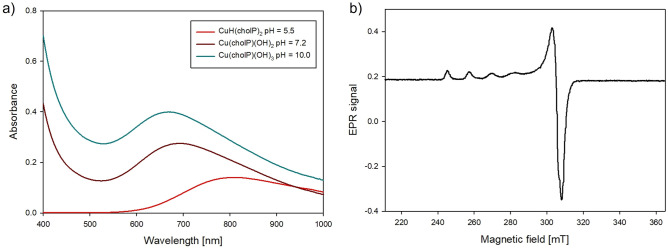
(**a**) UV-Vis spectra of complexes in Cu(II)/cholP 1:1 system, (**b**) EPR spectrum of CuH(cholP)_2_ complex.

On the basis of the analysis of UV-Vis (d–d transition energy), EPR (g_‖_ as well as A_‖_ parameters) and ^13^C and ^31^P NMR (changes in the chemical shifts between free ligand and ligand in complex), the coordination mode was specified. As reported in previous publications, there is a relationship between the type and number of donor atoms in the inner coordination sphere of Cu(II) complexes and UV-Vis, as well as the spectral parameters of EPR^32–34^. In the protonated form CuH(cholP)_2_ only one oxygen atom from the phosphate group is in the inner coordination sphere (λ_max_ = 801 nm, g_‖_ = 2.39, A_‖_ = 147 10^−4^ cm^−1^), (Table 2, Fig. 3). The active participation of the phosphate group in the complexation process is confirmed by a significant ^31^P NMR shift difference (Table 3). For the hydroxocomplexes Cu(cholP)(OH)_2_ and Cu(cholP)(OH)_3_ we observe decreasing values of λ_max_ as well as g_‖_ and an increasing value of A_‖_, which indicates the incorporation of more oxygen atoms into the inner coordination sphere. ^13^C NMR analysis showed no significant differences in shift values in the spectra of the free ligand versus the ligand in the complex. Only for the protonated form, small difference is observed (−0.12 ppm) as a result of noncovalent interactions between ligand molecules where the phosphate groups act as negative centres with the positively charged quaternary ammonium groups.

**Table 2:** Spectral parameters of UV-Vis and EPR studies for binary Cu(II)/cholP system.

Species	pH	*λ*_max_ (nm)	ε (dm^3^ mol^−1^ cm^−1^)	g_‖_	A_‖_ (10^−4^ cm^−1^)	Chromophore
Cu(HcholP)(cholP)	5.5	801	14.06	2.39	147	{1O}
Cu(cholP)(OH)_2_	7.2	694	28.85	2.36	153	{xO}
Cu(cholP)(OH)_3_	10.0	668	48.20	–	–	{xO}

**Table 3:** NMR differences between shifts for the free ligand and ligand in complex (ppm).

						cholP					Nucleoside or Nucleotide							
System	pH	C1	C2	C3	C3	C3	P	C2	C4	C5	C6	C1`	C2`	C3`	C4`	C5`	CH_3_	P
Cu(II)/cholP	5.5	− 0.02	− 0.12	0	0	0	− 9.91											
	7.2	0	− 0.02	0	0	0	− 1.16											
	10.0	− 0.01	0.01	0.01	0.01	0.01	0.10											
Cu(II)/cholP/Cyd	2.5	0	− 0.03	− 0.01	− 0.01	− 0	− 2.32	− 0.32	− 0.56	–	+ 0.20	− 0.17	+ 0.01	+ 0.05	+ 0.02	− 0.06		
	6.2	+ 0.01	− 0.13	− 0.01	0	0	− 6.95	+ 0.68	− 0.12	–	+ 0.05	− 0.43	− 0.04	− 0.05	− 0.09	− 0.02		
	8.0	+ 0.02	0	0	0	0	− 0.08	+ 0.26	0	− 0.34	+ 0.01	− 0.07	− 0.03	− 0.05	− 0.08	− 0.02		
Cu(II)/cholP/Thd	3.0	+ 0.18	+ 0.16	− 0.01	− 0.01	0	− 9.21	− 0.02	0.02	− 0.05	− 0.01	− 0.01	0.03	− 0.01	0	− 0.02	+ 0.01	
	6.5	− 0.01	− 0.12	− 0.01	0	0	− 9.49	− 0.02	+ 0.02	− 0.05	− 0.01	− 0.01	+ 0.03	− 0.01	0	− 0.02	+ 0.01	
	8.5	− 0.18	− 0.17	− 0.18	− 0.18	− 0.18	− 0.04	+ 0.11	+ 0.23	− 0.03	− 0.03	− 0.01	− 0.02	0	− 0.01	− 0.01	− 0.02	
Cu(II)/cholP/Urd	3.0	+ 0.01	0	0	0	0	− 2.31	+ 0.02	+ 0.04	− 0.03	− 0.02	+ 0.05	+ 0.01	− 0.04	− 0.04	− 0.04		
	6.7	− 0.01	− 0.05	− 0.01	0	0	− 5.29	− 0.57	0.78	–	− 0.02	− 0.48	− 0.02	− 0.03	− 0.06	− 0.02		
	7.7	0	0	− 0.01	0	− 0.01	− 0.14	− 0.36	− 0.11	− 0.12	+ 0.01	− 0.08	− 0.02	− 0.05	− 0.07	− 0.03		
	9.5	− 0.01	+ 0.02	0	0	0	− 0.03	+ 0.16	+ 0.33	− 0.03	− 0.07	+ 0.16	− 0.01	− 0.06	− 0.11	0		
Cu(II)/cholP/CMP	2.5	+ 0.02	− 0.03	0	+ 0.01	+ 0.03	− 2.55	+ 0.40	+ 0.05	–	+ 0.01	− 0.22	+ 0.04	+ 0.05	+ 0.14	− 0.04		− 2.08
	5.0	− 0.04	+ 0.06	0	0	0	− 2.53	+ 1.41	− 0.28	–	− 0.03	− 1.22	− 0.01	− 0.13	− 0.13	+ 0.03		+ 0.48
	6.0	+ 0.07	− 0.14	+ 0.01	+ 0.02	+ 0.02	− 3.49	+ 1.37	− 0.16	–	0	− 0.99	− 0.01	− 0.07	− 0.16	+ 0.06		− 2.84
Cu(II)/cholP/TMP	2.5	− 0.02	− 0.03	+ 0.01	+ 0.01	+ 0.02	− 2.40	− 0.01	− 0.03	− 0.01	− 0.01	− 0.01	+ 0.03	+ 0.02	+ 0.12	− 0.04	− 0.03	− 0.62
	5.6	− 0.01	+ 0.02	+ 0.01	+ 0.01	+ 0.01	− 5.33	− 0.03	+ 0.05	− 0.03	− 0.05	− 0.02	+ 0.07	+ 0.02	+ 0.02	+ 0.08	− 0.09	− 6.18
	6.5	+ 0.03	− 0.02	+ 0.01	+ 0.01	+ 0.01	− 2.51	− 0.09	+ 0.09	− 0.07	− 0.02	− 0.09	+ 0.05	+ 0.06	+ 0.07	+ 0.01	− 0.13	− 2.78
Cu(II)/cholP/UMP	4.7	+ 0.02	− 0.11	− 0.01	− 0.01	− 0.01	− 5.29	− 0.01	+ 0.04	− 0.06	− 0.07	− 0.08	+ 0.01	− 0.03	− 0.01	+ 0.04		− 5.04
	6.3	+ 0.03	− 0.06	0	0.0	0.01	− 4.91	− 0.23	+ 0.17	− 0.13	− 0.04	− 0.35	− 0.01	+ 0.03	+ 0.06	− 0.03		− 3.75

### Ternary Cu(II)/cholP/Nuc systems

#### Potentiometric studies of the Cu(II)/cholP/Nuc systems

The stability constants of the complexes created in the ternary Cu(II)/cholP/Nuc systems were determined taking into account the values for protonation and stability constants of the binary complexes Cu(II)/Nuc from our previous research and are given in Table 1. Figure 3 presents the distribution of species forming in the analyzed systems. The overall stability constants as well as equilibrium constants of all complexes are shown in Table 4.

**Table 4:** Overall and equilibrium stability constants of complexes in the Cu(II)/cholP/Nuc and Cu(II)/cholP/NMP systems (standard deviation is given in parenthesis).

Species	Overall stability constants logβ	Reactions	log*K*_*e*_
Cu(cholP)H_2_(Cyd)	27.37(4)	Cu^2+^ + (HcholP) + (HCyd) ⇆ Cu(cholP)H_2_(Cyd)	17.28
Cu(cholP)H(Cyd)	21.75(4)	Cu(Cyd) + (HcholP) ⇆ Cu(cholP)H(Cyd)	14.02
Cu(cholP)(Cyd)(OH)	8.19(4)	Cu(cholP)H(Cyd) + H_2_O ⇆ Cu(cholP)(Cyd)(OH) + 2H^+^	0.21
Cu(cholP)H_2_(Thd)	26.34(5)	Cu^2+^ + (HcholP) + (HThd) ⇆ Cu(cholP)H_2_(Thd)	11.07
Cu(cholP)(Thd)	13.56(5)	Cu(Thd) + (cholP) ⇆ Cu(cholP)H(Thd)	7.89
Cu(cholP)(Thd)(OH)	6.85(4)	Cu(cholP)(Thd) + H_2_O ⇆ Cu(cholP)(Thd)(OH) + H^+^	7.06
Cu(cholP)H_2_(Urd)	24.49(4)	Cu^2+^ + (HcholP) + (HUrd) ⇆ Cu(cholP)H_2_(Urd)	9.79
Cu(cholP)(Urd)	11.23(4)	Cu^2+^ + (cholP) + (Urd) ⇆ Cu(cholP)(Urd)	11.23
Cu(cholP)(Urd)(OH)	4.50(4)	Cu(cholP)(Urd) + H_2_O ⇆ Cu(cholP)(Urd)(OH) + H^+^	7.04
Cu(cholP)(Urd)(OH)_2_	− 4.25(4)	Cu(cholP)(Urd)(OH) + H_2_O ⇆ Cu(cholP)(Urd)(OH)_2_ + H^+^	5.02
Cu(cholP)H_3_(CMP)	28.70(5)	Cu^2+^ + (HcholP) + (H_2_CMP) ⇆ Cu(cholP)H_3_(CMP)	12.32
Cu(cholP)H_2_(CMP)	24.11(4)	Cu^2+^ + (HcholP) + (HCMP) ⇆ Cu(cholP)H_2_(CMP)	12.21
Cu(cholP)H(CMP)	18.59(4)	(HcholP) + Cu(CMP) ⇆ Cu(cholP)H(CMP)	10.40
Cu(cholP)(CMP)(OH)	5.83(3)	Cu(CMP)(OH) + (cholP) + H_2_O ⇆ Cu(cholP)(CMP)(OH) + H^+^	3.12
Cu(cholP)H_3_(TMP)	32.50(3)	Cu^2+^ + (HcholP) + (H_2_TMP) ⇆ Cu(cholP)H_3_(TMP)	11.25
Cu(cholP)H_2_(TMP)	27.46(3)	(HcholP) + Cu(HTMP) ⇆ Cu(cholP)H_2_(TMP)	8.40
Cu(cholP)H(TMP)	21.13(3)	(cholP) + Cu(HTMP) ⇆ Cu(cholP)H(TMP)	7.56
Cu(cholP)(TMP)(OH)	7.63(3)	(cholP) + Cu(TMP)(OH) ⇆ Cu(cholP)(TMP)(OH)	7.24
Cu(cholP)H_2_(UMP)	24.52(4)	Cu^2+^ + (HcholP) + (HUMP) ⇆ Cu(cholP)H_2_(UMP)	9.54
Cu(cholP)H(UMP)	18.33(5)	Cu^2+^ + (cholP) + (HUMP) ⇆ Cu(cholP)H(UMP)	8.83
Cu(cholP)(UMP))OH)	5.31(4)	(cholP) + Cu(UMP) + H_2_O ⇆ Cu(cholP)(UMP)(OH) + H^+^	13.05
Cu(cholP)(UMP)(OH)_2_	− 3.86(5)	Cu(cholP)(UMP)(OH) + H_2_O ⇆ Cu(cholP)(UMP)(OH)_2_ + H^+^	4.60

In the ternary system containing copper(II) ions, phosphocholine and cytidine, three types of ternary complexes were found: protonated MLL`H_2_, MLL`H and hydroxocomplex MLL`(OH). In the end of measurement, binary Cu(cholP)(OH)_3_ complex occurs in small amount. The first protonated complex Cu(cholP)H_2_(Cyd) exists in the solution from the beginning of the measurement, binding 100% of copper(II) ions. At pH close to 4.0, stepwise deprotonation takes place, and the monoprotonated form starts to create and becomes the dominant form at pH 6.2 binding almost 80% of the copper ions. From pH 6.0 to 11.0 Cu(cholP)(Cyd)(OH) is observed and from pH 7.5 to 10.0 binds almost 100% Cu(II).

**Figure 4:**
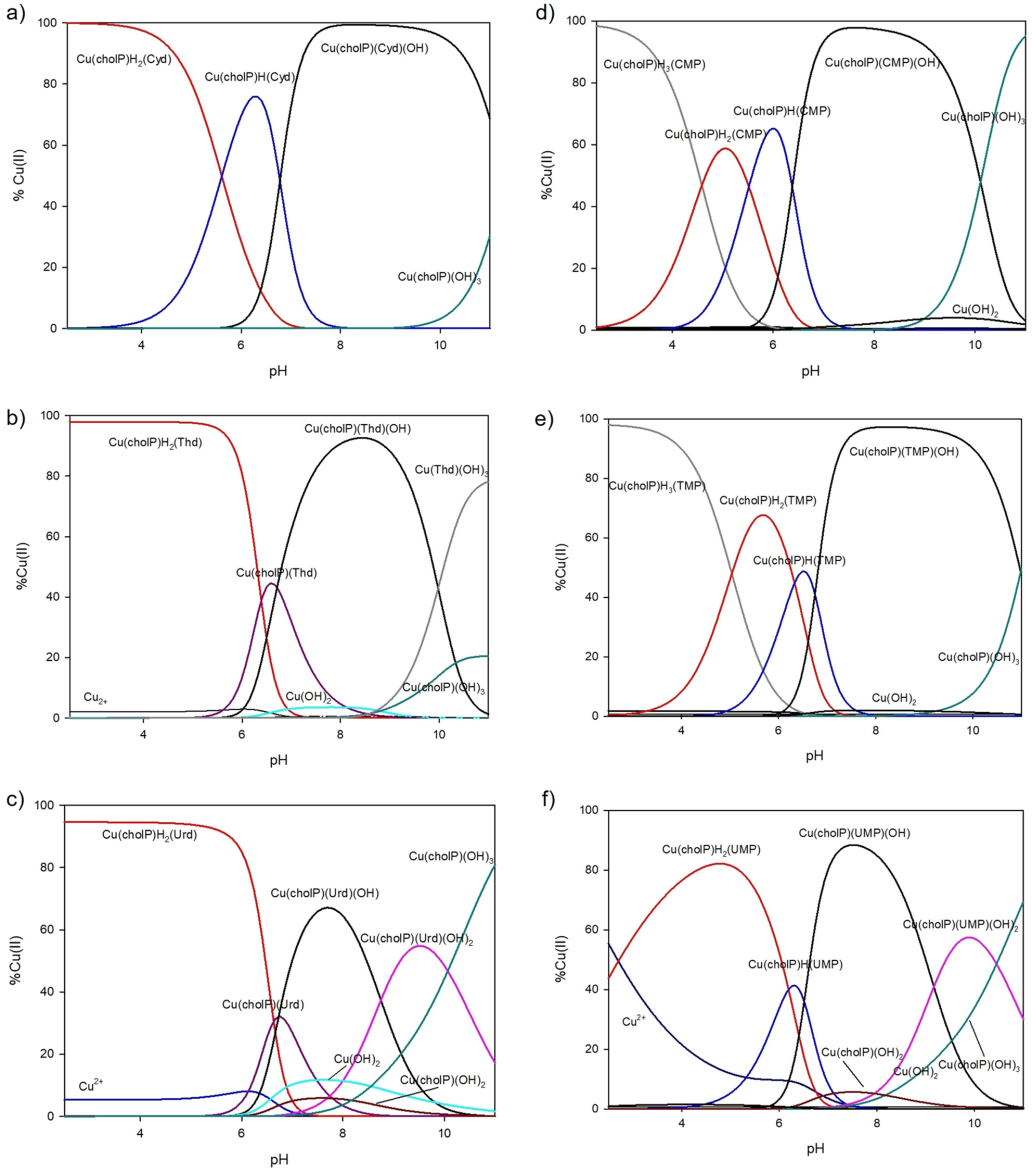
Distribution diagrams for (**a**) Cu(II)/cholP/Cyd (**b**) Cu(II)/cholP/Thd (**c**) Cu(II)/cholP/Urd (**d**) Cu(II)/cholP/CMP (**e**) Cu(II)/cholP/TMP and (**f**) Cu(II)/cholP/UMP systems.

In the Cu(II)/cholP/Thd system, complexes Cu(cholP)H_2_(Thd), Cu(cholP)(Thd) and Cu(cholP)(Thd)(OH) were found (stability constants are given in Table 4). The protonated form (log*K*_*e*_ = 11.07) occurred up to a pH close to 7.0 and bound almost 100% of copper(II) ions from pH 2.5 to 6.0. At pH 6.5 a simple MLL` type complex binds approximately 40% of copper ions. Between pH values 5.0 and 8.5 it overlaps with Cu(cholP)H_2_(Thd) and Cu(cholP)(Thd)(OH) complexes (Fig. 4). The latter ternary complex in this system binds more than 90% of metal ions at pH 8.5 where binary hydroxocomplexes Cu(cholP)(OH)_3_ and Cu(Thd)(OH)_3_ start forming.

The results of the potentiometric titrations and computer data analysis indicate that the following complexes are formed in the ternary Cu(II)/cholP/Urd system: Cu(cholP)H_2_(Urd), Cu(cholP)(Urd), Cu(cholP)(Urd)(OH), Cu(cholP)(Urd)(OH)_2_ as the well as binary hydroxocomplexes Cu(cholP)(OH)_2_ and Cu(cholP)(OH)_3_ (Fig. 4). At the acidic environment, the distribution diagram looks similar to that one containing thymidine: deprotonated form dominates from pH 2.5 to 6.0 binding almost total amount of copper(II) introduced into solution. Subsequently, from pH 5.5 to 8.5 the simple MLL` type of complex is overlapped by protonated one and the hydroxocomplex. The overall stability constants of the type of complexes in the ternary system containing uridine are slightly lower compared to the ternary system containing thymidine; the same tendency is observed for complexes in the binary Cu(II)/Urd and Cu(II)/Thd systems (Tables 1 and 4). In the Cu(II)/cholP/Thd system, the second ternary hydroxocomplex Cu(cholP)(Urd)(OH)_2_ exists. Its maximum relative concentration, almost 60%, occurs at pH ~ 9.5. The complex Cu(cholP)(OH)_3_ appears at pH close to 8.0 and binds 80% of Cu(II) at pHs of about 11.0 an outside the scope of the study.

#### Spectroscopic studies of the Cu(II)/cholP/Nuc systems

Spectroscopic studies were recorded at the pH values of individual species except for complexes that overlapped with other forms to a significant extent. As in the case of the binary system described above, UV-vis, EPR, carbon and phosphorus NMR, FT–IR, and, in addition, CD measurements were carried out.

**Figure 5:**
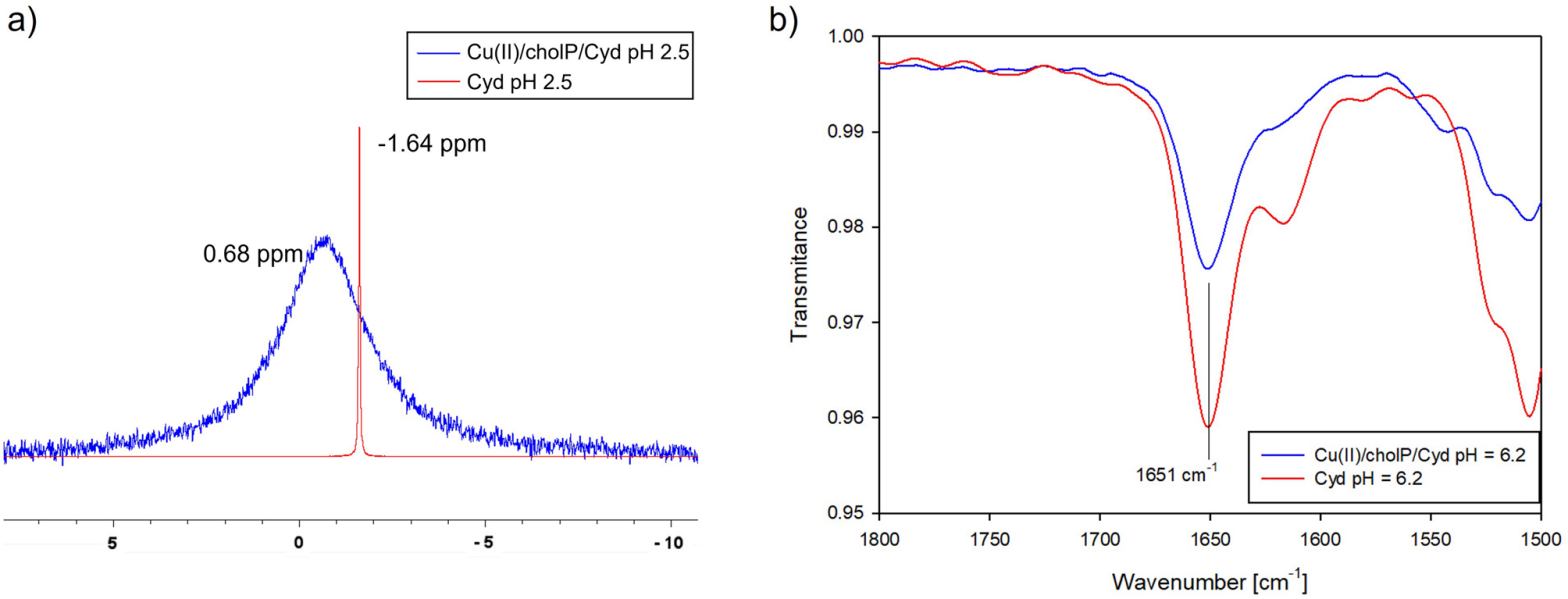
^31^P NMR spectra of ligand and ligand in Cu(cholP)H_2_(Cyd) complex, IR spectra of Cu(cholP)H(Cyd) complex compared to the free Cyd at the same pH.

For the Cu(cholP)H_2_(Cyd) complex, λ_max_ = 799 nm and EPR parameters are respectively g_‖_ = 2.40 and A_‖_ = 138 10^−4^ cm^−1^. Their values indicate the presence of only one oxygen atom derived from the phosphate group of phosphocholine (shift between free ligand and ligand in the complex in ^31^P NMR is  − 2.32 ppm (Fig. 5). The changes in the chemical shifts in ^13^C NMR (C(2)  − 0.32 ppm, C(4)  − 0.56 ppm) point to weak interactions between endocyclic nitrogen atom N(3) and phosphocholine. For the next form, which is dominant at pH 6.2, the value of λ_max_ as well as g_‖_ decrease and a value of A_‖_ increase (Table 5). This testifies with considerable shifts between free ligand and ligand in complex in ^31^P and ^13^C NMR, that in the inner coordination sphere, there are one nitrogen atom (N(3) of Cyd) and an oxygen atoms of the phosphate group of cholP. Due to significant differences in shifts on carbons C(2) and C(4) FT–IR studies were performed. The positions of the stretching vibration bands assigned to the carbonyl groups (1651 cm^−1^ for the ligand and complex both) testify to the lack of interactions of these groups of the bioligands with copper(II) ions in the whole pH range considered (Fig. 5). The activity of the phosphate group was confirmed not only by changes in ^31^P NMR but also by IR spectra, which show small changes in the positions of the antisymmetric stretching bands (1093 cm^−1^ in the spectrum of free ligand and 1096 cm^−1^ for complex)^35^. For Cu(cholP)(Cyd)(OH), spectral parameters suggest the same coordination mode, with additional oxygen atoms in the inner coordination sphere.

**Table 5:** Spectral parameters from UV-Vis and EPR studies for investigated ternary systems.

Species	pH	λ_max_ (nm)	ε (dm^3^ mol^−1^ cm^−1^)	g_‖_	A_‖_ (10^−4^ cm^−1^)	Chromophore
Cu(cholP)H_2_(Cyd)	2.5	799	10	2.40	138	{1O}
Cu(cholP)H(Cyd)	6.2	764	18	2.34	165	{1N, xO}
Cu(cholP)(Cyd)(OH)	8.0	670	56	2.32	166	{1N, xO}
Cu(cholP)H_2_(Thd)	4.0	800	13.96	2.39	143	{1O}
Cu(cholP)(Thd)(OH)	8.5	686	28.81	2.36	154	{1N, xO}
Cu(cholP)H_2_(Urd)	4.0	804	13.82	2.39	139	{1O}
Cu(cholP)(Urd)(OH)	7.7	634	90.79	2.36	157	{1N, xO}
Cu(cholP)(Urd)(OH)_2_	9.5	626	91.85	–	–	
Cu(cholP)H_3_(CMP)	2.5	797	12.24	2.40	137	{1O}
Cu(cholP)H_2_(CMP)	5.0	794	13.72	2.36		{ xO}
Cu(cholP)H(CMP)	6.0	779	26.06	–	–	{1N, xO}
Cu(cholP)(CMP)(OH)	7.5	680	79.66	–	–	
Cu(cholP)H_3_(TMP)	2.5	797	14.57	2.39	136	{1O}
Cu(cholP)H_2_(TMP)	5.6	797	16.60	2.34	141	{ xO}
Cu(cholP)H(TMP)	6.5	732	36.35	–	–	{1N, xO}
Cu(cholP)(TMP)(OH)	7.5	680	64.97	–	–	{1N, xO}
Cu(cholP)H_2_(UMP)	4.7	807	15.76	2.38	143	{1O}
Cu(cholP)H(UMP)	6.3	765	29.98	–	–	{ xO}
Cu(cholP)(UMP))OH)	7.5	691	69.42	–	–	{1N, xO}
Cu(cholP)(UMP)(OH)_2_	9.8	670	73.53	–	–	{1N, xO}

In the Cu(II)/cholP/Thd system, for the first complex the energy of d–d transition, , λ_max_ = 800 nm, taken at pH 4.0, suggests the involvement of only one oxygen atom. The EPR parameters g_‖_ = 2.39 and A_‖_ = 143 10^−4^ cm^−1^, as well as the change in ^31^P NMR between shifts, are in accordance with this and point to creating a molecular complex ML···L` type where thymidine is outside the inner coordination sphere of Cu(II) ions and interacts non—covalently with the anchor binary complex of copper ions with phosphocholine. Confirmation of this assumption was also IR spectra of ligand and complex where the positions of the antisymmetric stretching band shifted slightly (1085 cm^−1^ for the cholP and 1088 cm^−1^ for complex). For the hydroxocomplex species in this system, the spectral parameters change to a considerable extent, suggesting the involvement of the nitrogen atoms N(3) of thymidine molecules in the complexation process.

As in the previous system, for the first species that is created in the Cu(II)/cholP/Urd system we observed a molecular complex in which the copper ions bond through the oxygen atom of the phosphate group of cholP (λ_max_ = 804 nm, g_‖_ = 2.39 and A_‖_ = 139 10^−4^ cm^−1^, ^31^P Δδ = −2.31 ppm). The antisymmetric stretching band slides from 1085 cm^−1^ for the ligand to 1087 cm^−1^ for complex. Based on the changes in the UV-vis and EPR spectral parameters values (Table 5), we can see that the incorporation of the endocyclic nitrogen atom N(3) from uridine into the internal coordination sphere occurs at pH 7.7 where the first hydroxocomplex dominates. This is confirmed by the changes in the chemical shifts on C(2) and C(4) atoms (−0.36 and  − 0.11 ppm respectively) between free ligand and ligand in the complex. The maximum absorption for the Cu(cholP)(Urd)(OH)_2_ complex decreases slightly from 634 nm to 626 nm which is caused by the inclusion of an additional oxygen atom in the inner coordination sphere.

#### Circular dichroism studies of the Cu(II)/cholP/Nuc systems

In the ternary Cu(II)/cholP/Nuc systems chiral components were added into solutions. Since the direct analysis of ternary systems is very difficult because the CD spectrum shows the sum of all interactions, it was first necessary to measure the spectra of binary systems in order to determine the influence of the third component when studying ternary systems. For this purpose, the spectra of the Cu(II)/Nuc and cholP/Nuc binary systems were measured at pH previously determined by potentiometric measurements.

Measurements made for the Cu(II)/Cyd system indicate that the basic pattern of Cotton effects in the studied range remains unchanged. In an acidic solution, the ∆ε maximum is shifted towards longer wavelengths, which corresponds to a change in the absorption maximum of about 10 nm (ε_max_ 279 for pH = 3.0, 271 for pH = 6.2, 270 for pH = 8.0). This is due to the presence of the protonated form of the pyrimidine base, which affects the change in the energy of electronic transitions in the aromatic ring.

An analogous situation can be observed for the cholP/Cyd system, which indicates that in the tested solutions the shape of the CD spectrum is mostly pH dependent.

**Figure 6:**
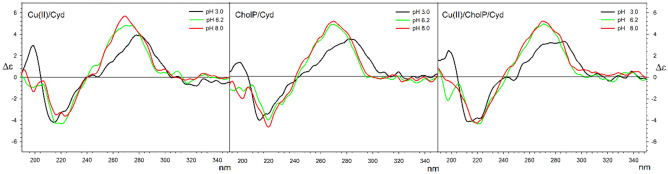
CD spectra for Cu(II)Cyd (left); cholP/Cyd (middle) and Cu(II)/cholP/Cyd (right) systems in water solutions at C = 2*10^−4^ mol*dm^−1^.

With the results of the above discussed binary systems in hand, a series of CD spectra of ternary systems was measured at corresponding pH. Also in this cases, no significant changes in the CD spectrum were observed, which indicates that the formation of complexes observed for particular pHs does not significantly affect their conformations (Fig. 6). All above discussed data for Cytidine are presented in Table 6. Tabular data and spectra for analogous systems with thymidine and uridine are presented in Supplementary materials (Figs. S12–S13, Tables S1, S2).

**Table 6:** Cotton effects maxima for Cu(II)/Cyd, cholP/Cyd and Cu(II)/cholP/Cyd complexes in water solutions at C = 2*10^−4^ mol*dm^−1^.

	Δε [nm]		
	pH 3.0	pH 6.2	pH 8.0
Cu(II)/Cyd	3.92 (278)	4.86 (275)	5.69 (270)
		4.86 (270)	
	− 4.21 (215)	− 4.32 (221)	− 3.63 (224)
			− 3.50 (217)
	2.94 (199)	− 0.93 (200)	− 1.35 (199)
cholP/Cyd	3.57 (282)	4.97 (269)	5.21 (270)
	− 4.01 (213)	− 3.96 (220)	− 4.65 (220)
	1.39 (198)	− 1.26 (201)	− 2.05 (220)
		− 1.20 (193)	
Cu(II)/cholP/Cyd	3.33 (287)		
	3.21 (276)	4.95 (271)	5.23 (271)
	− 3.82 (222)	− 4.35 (221)	− 4.28 (220)
	− 4.12 (213)	− 4.14 (216)	
	2.51 (198)	− 2.17 (197)	

### Ternary Cu(II)/cholP/NMP systems

#### Potentiometric studies of the Cu(II)/cholP/NMP systems

For Cu(II)/cholP/NMP systems, computer—aided analysis of the potentiometric titration data revealed the formation of ternary protonated species MLL`H_x_ together with hydroxocomplexes MLL`(OH)_x_.

With the beginning of the titration for system Cu(II)/cholP/CMP, we see dominance in almost one hundred percent of the triprotonated form (Fig. 4). The formation of Cu(cholP)H_2_(CMP) complex begins at pH about 2.5 and vanishes at pH close to 7.0. The maximum amount of Cu(II) complexing for this individual occurs at pH 5.0 (60%). In the pH range between 4.0 and 7.5 we observe a monoprotonated form, which at pH close to 6.0 binds 65% of copper ions in the examined solution. The last ternary form for this system is monohydroxocomplex which binds more than 90% of metal ions in wide pH range (6.5–9.5). The successive stability constants of the ternary complexes are log*K*_*e*_ = 12.32, log*K*_*e*_ = 12.21, log*K*_*e*_ = 10.40 and log*K*_*e*_ = 3.12 respectively (Table 4) From pH 10.0 the dominant form is the binary Cu(cholP)(OH)_3_ complex, the concentration of which increases until the end of the tested pH range.

The same types of complexes as in the system described above were observed in the ternary Cu(II)/cholP/TMP. Analogous protonated complex species form and dominate at slightly higher pH values: Cu(cholP)H_3_(TMP) dominates also at the beginning of the measurement, Cu(cholP)H_2_(TMP) at pH 5.5 (70%), Cu(cholP)H(TMP) at pH 6.5 (50%). The Cu(cholP)(TMP)(OH) form binds almost to the total amount of Cu^2+^ in the pH range 7.0–10.0.

In the Cu(II)/cholP/UMP system, Cu(cholP)H_2_(UMP), Cu(cholP)H(UMP), Cu(cholP)(UMP)(OH) and Cu(cholP)(UMP)(OH)_2_ species were detected. At the beginning of the measurement, 55% of free copper(II) ions and 45% bound in the Cu(cholP)H_2_(UMP) complex were observed. The first protonated form exists in the system up to a pH close to 7.0 and reaches its maximum concentration (80%) at pH 4.7. The monoprotonated form between pH 4.0 and 7.5 overlaps. The formation of Cu(cholP)(UMP)(OH) starts at pH close to 6.0 and dominates at pH 7.5 binding 90% of copper(II) ions. Cu(cholP)(UMP)(OH)_2_ exists in the investigated solution from pH more than 7.0 to the end of the tested pH range. Again, at alkaline pH we observe binary Cu(cholP)(OH)_3_ which reaches maximum concentration an outside the scope of the study.

#### Spectroscopic studies of the Cu(II)/cholP/NMP systems

The λ_max_ of 797 nm for Cu(cholP)H_3_(CMP) points to the monofunctional coordination of type 1O which is in agreement with the EPR spectral parameters (g_‖_ = 2.40 and A_‖_ = 137 10^−4^ cm^−1^) (Figs. S1, S2). Chemical shifts between ligand and ligand in the complex on the ^31^P NMR spectra are significant for cholP and CMP also (−2.55 ppm and  − 2.08 ppm respectively). Taking into consideration values of the protonation constants log*K*_*e*_ = 5.48 and 4.48 for cholP and CMP, respectively, we concluded that in the inner coordination sphere, only the oxygen atoms of phosphate group of CMP are involved, similar like for Cu(enP)H_4_(CMP)^30^. Some changes between shifts in ^13^C NMR on C(2) and C(4) atoms and positions of the antisymetric stretching bands N–CH_3_ from –N^+^(CH_3_)_3_ point to weak interactions between the ring of CMP as negative center and positively charged phosphocholine (Figs. S3–S11)^36^. For Cu(cholP)H_2_(CMP) the λ_max_ and g_‖_ slightly decrease, indicating the incorporation of another oxygen atom into the internal coordination sphere, this time from cholP (Table 4). The spectra of ^13^C NMR show some changes between shifts between free CMP and CMP in complex for C(2) and C(4) as a result of weak interactions between bioligands where CMP acts as a negative center and positively charged phosphocholine molecules (Table 3).

For Cu(cholP)H(CMP) EPR spectrum was not able to record because of the precipitation. However, the value of λ_max_ in UV-Vis studies decreases to 779 nm and we can assume that the endocyclic nitrogen atom N(3) of CMP also participates in the complexation process at pH 6.0. The significantly reduced value of λ_max_ parameter for the complex indicates the participation of both nitrogen and oxygen in the complexation process of copper ions. Due to the formation of a precipitate in samples made in the required concentration to perform NMR studies, we could not confirm it with this spectroscopic method.

For the Cu(cholP)H_3_(TMP) and Cu(cholP)H_2_(TMP) species, the spectral parameters indicate the same type of coordination as for analogous forms of the CMP—containing system. For Cu(cholP)H_3_(TMP) difference in shifts in ^31^P NMR points to the involvement of phosphate group of TMP in complexation. For the dominant in pH range 7.0–10.0 complex, the value of λ_max_ declines significantly to 680 nm and this is a sign of the internal coordination sphere which includes both nitrogen and oxygen atoms of the tested bioligands.

In the inner coordination sphere of the protonated complex in Cu(II)/cholP/UMP, the system spectral parameters suggest the activity of the phosphate group of the cholP and the absence of activity of the endocyclic nitrogen atom N(3) and the UMP phosphate group (Tables 3 and 5). For both hydroxocomplexes, the values of λ_max_ (691 nm and 670 nm for Cu(cholP)(UMP)(OH) and Cu(cholP)(UMP)(OH)_2_ respectively) point to the activity of nitrogen N(3) and oxygen atoms.

#### Circular dichroism studies of the Cu(II)/cholP/NMP systems

Analogous measurements of circular dichroism spectra were measured for the corresponding ternary systems containing CMP, UMP and TMP nucleotides. In systems of this type, the phosphate residue may behave as an electron donor, taking part in the formation of complex compounds, which may affect the conformation of nucleotides. Such conformational changes can in turn be observed in the CD spectra, or if similar spectra are obtained, it can be concluded that there are no substantial conformational changes. Also in the case of systems containing nucleotides, the pH of the measured solutions was selected on the basis of previously performed potentiometric measurements.

**Figure 7:**
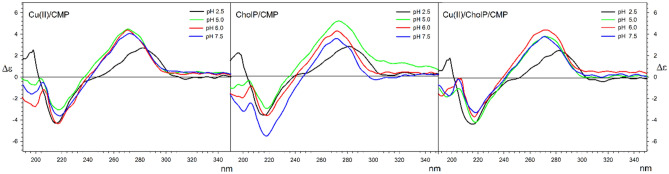
CD spectra for Cu(II)CMP (left); cholP/CMP (middle) and Cu(II)/cholP/CMP (right) systems in water solutions at C = 2*10^−4^ mol*dm^−1^.

Measurements made for the Cu(II)/CMP and cholP/CMP systems indicate that also in these cases the basic patterns of Cotton effects in the studied range remains unchanged. Analogous situations were observed for systems with TMP and UMP [see SM Figs. S14–S15, Tables S3, S4)]. With the results of NMP binary systems in hand, a series of CD spectra of ternary systems was measured at corresponding pH. Also in this case, no significant changes in the CD spectrum were observed, which indicates that the formation of complexes observed for particular pHs does not significantly affect their conformations and the influence of the phosphate residue on conformational changes is not significant (Fig. 7). All above discussed data for CMP systems can be seen in Table 7.

**Table 7:** Cotton effects maxima for Cu(II)/CMP, cholP/CMP and Cu(II)/cholP/CMP complexes in water solutions at C = 2*10^−4^ mol*dm^−1^.

	Δε [nm]			
	pH 2.5	pH 5.0	pH 6.0	pH 7.5
Cu(II)/CMP	2.71 (282)	4.49 (272)	4.38 (271)	4.05 (272)
	− 4.31 (216)	− 3.05 (219)	− 4.36 (218)	− 3.62 (219)
	2.52 (198)	− 0.77 (200)	− 2.77 (200)	− 1.58 (197)
cholP/CMP	2.85 (281)	5.46 (274)	4.30 (272)	2.90 (272)
	− 3.55 (216)	− 2.69 (218)	− 3.60 (218)	− 6.19 (218)
	2.52 (198)	− 0.53 (199)	− 1.94 (200)	− 3.90 (201)
		− 0.82 (192)		
Cu(II)/cholP/CMP	2.46 (283)	3.78 (271)	4.51 (273)	3.77 (272)
	− 4.39 (216)	− 4.23 (18)	− 3.58 (218)	− 3.34 (219)
	1.77 (199)	− 1.87 (196)	− 1.64 (193)	− 1.77 (198)

Juxtaposing the CD spectra of systems containing nucleosides and nucleotides, it can be seen that the greatest changes can be observed for solutions with low pH in the short—wave range (around 200 nm), where the absorption of the phosphate residue also occurs. However, comparing the spectra of nucleoside complexes and nucleotides with and without cholP, it can be stated that these changes also occur for copper—nucleotide complexes, which clearly indicates that they are the result of protonation of pyrimidine bases and not phosphate residues in nucleosides and in cholP.

Comparing these changes with the shift of the long wavelength Cotton effects at low pH, associated with the absorption of pyrimidine bases, it can be concluded that the protonation of these bases has a significant impact on the conformations of these compounds, regardless of the presence of other components of the solutions, whose influence is much smaller.

## Conclusions

In all ternary tested systems containing copper(II) ions, phosphocholine and pyrimidine nucleosides, MLL`H_2_ and MLL`(OH) complexes were established. Monoprotonated form is observed only for system containing cytidine, simple MLL` type of complexes for CU(II)/cholP/Thd and Cu(II)/cholP/Urd. The MLL`(OH)_2_ complex is only found in systems containing Urd and UMP, which shows their greater tendency and stability to form hydroxocomplexes in both the ternary and in the binary systems with copper ions. In the ternary systems containing monophosphorylated nucletides, the formation of MLL`H_2_, MLL`H and MLL`(OH) complexes has been established as well as MLL`H_3_ for Cu(II)/cholP/CMP and Cu(II)/cholP/TMP.

Comparing the distribution of forms for all three systems containing nucleosides, we can see that for systems with uridine and thymidine, the curves look quite similar. The distribution diagram for the system with cytidine looks different: The second complex that is created is monoprotonated and dominates, in contrast to the simple complexes in Cu(II)/cholP/Urd and Cu(II)/cholP/Thd. According to the spectral parameters, we can observe the earlier deprotonation of N(3) and the inclusion of nitrogen atoms in the inner coordination sphere. This shows clear differences in the acid–base properties of these ligands, which are already reflected in the stage of differences in the values of the protonation constants. It was observed that the cholP phosphate group is actively involved in the complexation of copper ions over the entire pH range studied, even after the inclusion of nucleoside nitrogen atoms. It has been confirmed by both nuclear magnetic resonance spectroscopy and FT–IR studies.

For ternary systems containing nucleotides, distribution diagrams of Cu(II)/cholP/CMP and Cu(II)/cholP/TMP look similar and different than for Cu(II)/cholP/UMP. This is the only system in which the MLL`H_3_ form is not present and free copper ions are observed at the beginning of the potentiometric titration. Also, it is the only one of the three systems where the MLL`(OH)_2_ form has been recorded. This indicates a tendency to form stable ternary complexes at slightly higher pH values. The preference for the formation of hydroxocomplexes was observed for the binary Cu(II)/UMP system. Another noteworthy difference for the Cu(II)/cholP/UMP system is the fact that the complex is first formed by binding the metal through the phosphate group of the phosphocholine, and the weak interactions between the bioligands and the incorporation of the phosphate group of the nucleotide occurs at a slightly higher pH value. On the basis of ^13^C as well as ^31^P NMR and FT–IR studies, it was found that for the Cu(II)/cholP/CMP and Cu(II)/cholP/TMP systems, oxygen atoms located in the internal coordination sphere come from the appropriate nucleotide.

Potentiometric and spectroscopic studies described above provide information about the binding affinity between phosphocholine and nucleotides. By determining the stability constants or dissociation constants of the complexes, we can assess the strength of the interaction. This knowledge may be crucial for understanding the molecular recognition processes that occur in biological systems, such as enzyme–substrate interactions or protein–ligand binding. Understanding the spatial arrangement and intermolecular interactions within these complexes can shed light on their functional roles in biological processes. It can also aid in drug design and development by providing insights into potential binding sites and interactions. In summary, potentiometric and spectroscopic results on described complexes provide valuable insights into their binding affinity, structure, cellular signaling, biochemical pathways, and drug interactions. Such information enhances our understanding of biological systems and has implications for various fields, including biochemistry, pharmacology, and medicine.

## Methods

Phosphocholine chloride calcium salt tetrahydate, cytidine, cytidine 5`monophosphate, uridine 5`monophosphate disodium salt (from Sigma Aldrich: Steinheim am Albuch Baden–Württemberg, Germany), thymidine 5`-monophosphate disodium salt (from Alfa Aesar: Thermo Fisher, Kandel, Germany), uridine (from Merck), and thymidine (from Fluka Chemie GmbH: Honeywell Research Chemicals, Buchs, Switzerland) were used without additional purification. Copper(II) nitrate (from Merck) was purified by recrystallization from water and the concentration of copper(II) ions in the prepared solution was determined by the method of Inductively Coupled Plasma Optical Emission Spectrometry (ICP OES) (Shimadzu, Kyoto, Japan). All the prepared solutions and measurements performed were carried out using demineralized, carbonate–free water.

Potentiometric titrations were performed using a Methrom system (Titrino 702 equipped with an autoburette with a combined glass electrode). Each time before starting a series of measurements, the pH metre was calibrated with two standard buffer solutions and the electrode was calibrated in terms of H^+^ ions^37^. The concentration of Cu(II) and ligands were 1 × 10^−3^ M and the ratio between metal:ligand in binary system was 1:1 and 1:1:1 metal:ligand:ligand` in ternary systems. All measurements were carried out under strict conditions: temperature of 20 ± 1 °C, ionic strength of µ = 0.1 M (KNO_3_), in a helium atmosphere (He 5.0), using CO_2_–free NaOH (0.1922 M) in the pH range between 2.5 and 11.0. At least 12 titrations for each system were performed with 150–350 points for each titration. Hyperquad 2020 program was used to determine the protonation constants and stability constants of the complexes as described before^27,30,38^.

Samples for the spectroscopic studies were prepared in the pH of domination of each form. For UV-Vis, samples were performed in H_2_O in ratio 1:1 for binary and 1:1:1 for ternary systems. The concentration was 0.001–0.02 M. Spectra were recorded on an Evolution 300 UV-Vis ThermoFisher Scientific spectrometer (xenon lamp, range 450–950 nm, accuracy 0.2 nm, sweep rate 120 nm/min) in PLASTIBRAND PMMA cell with 1 cm path length at room temperature.

Water : glycol mixture in ratio 3:1 was used to prepare samples for EPR measurements. Spectra were recorded on the SE/X 2547 Radiopan instrument at  − 196 °C using capillary tubes (130 µm^3^).

For the ^13^C and ^31^P NMR measurements, as well as FT–IR, samples were performed in D_2_O and pD was adjusted by NaOD and DCl, taking into account that pD = pH + 0.4^39^. For FT–IR the measurements were collected with the ATR technique in the range of 400–4000 cm^−1^ using INVENIO R (Bruker). NMR spectra were recorded on an AVANCE III Bruker 500 MHz spectrometer in the concentration of ligand 0.1 M and the ratio M:L or M:L:L` 1:100 and 1:100:100 respectively using dioxane as an internal standard.

CD and corresponding UV spectra were recorded on JASCO J810 spectropolarimeter at room temperature. The spectra were recorded in the range of 190–400 nm in water solutions and were accumulated with 8 scans for all complexes studied. Water for the experiments was extra purified by Merck Milli–Q apparatus to lower the absorbance especially in the short–wave part of measuring range. The measurements were performed in N_2_ gas atmosphere (flow 10 L/min) and optical pathlength was 0.1 cm. Concentrations of measured solutions were of 2 × 10^−4^ that allowed to keep both the absorbance and noise at an acceptable level.

## Supplementary Information

Supplementary Information.

## Data Availability

All data generated or analyzed during this study are included in this published article (and its supplementary information file).

## Abbreviations

cholP: Phosphocholine
PC: Phosphatidylcholine
Nuc: Nucleosides
NMP: Monoprosphorylated nucleotide/nucleoside
CRP: C-reactive protein
Cyd: Cytidine
CMP: Cytidine-5`-monophosphate
Thd: Thymidine
TMP: Thymidine-5`-monophosphate
Urd: Uridine
UMP: Uridine-5`-monophosphate
Uv-Vis: Ultraviolet-visible spectroscopy
EPR: Electron paramagnetic resonance
NMR: Nuclear magnetic resonance
FT-IR: Fourier-transform infrared spectroscopy
CD: Circular dichroism
