# Supplementary material for: Biocoordination reactions in copper(II) ions and phosphocholine systems including pyrimidine nucleosides and nucleotides
Source: Sci Rep. 2023 Jul 4;13:10787. doi: 10.1038/s41598-023-37986-1 (PMC10319719; doi:10.1038/s41598-023-37986-1)
Supplement: Supplementary file 1 — Supplementary Information. [file 41598_2023_37986_MOESM1_ESM.docx]

**Supplementary** **materials**

**Biocoordination reactions in copper(II) ions and phosphocholine systems including pyrimidine nucleosides and nucleotides**

Malwina Gabryel-Skrodzka, Martyna Nowak, Jakub Grajewski and Renata Jastrzab

The set of EPR, UV-Vis, ^13^C NMR, ^31^P NMR, IR spectra of the Cu(cholP)H_3_(CMP) complex as well as corresponding to the free ligand ^13^C NMR, ^31^P NMR, IR spectra at the same pH and results from CD spectroscopy.

**Cu(cholP)H_3_(CMP) pH=2.5**

**
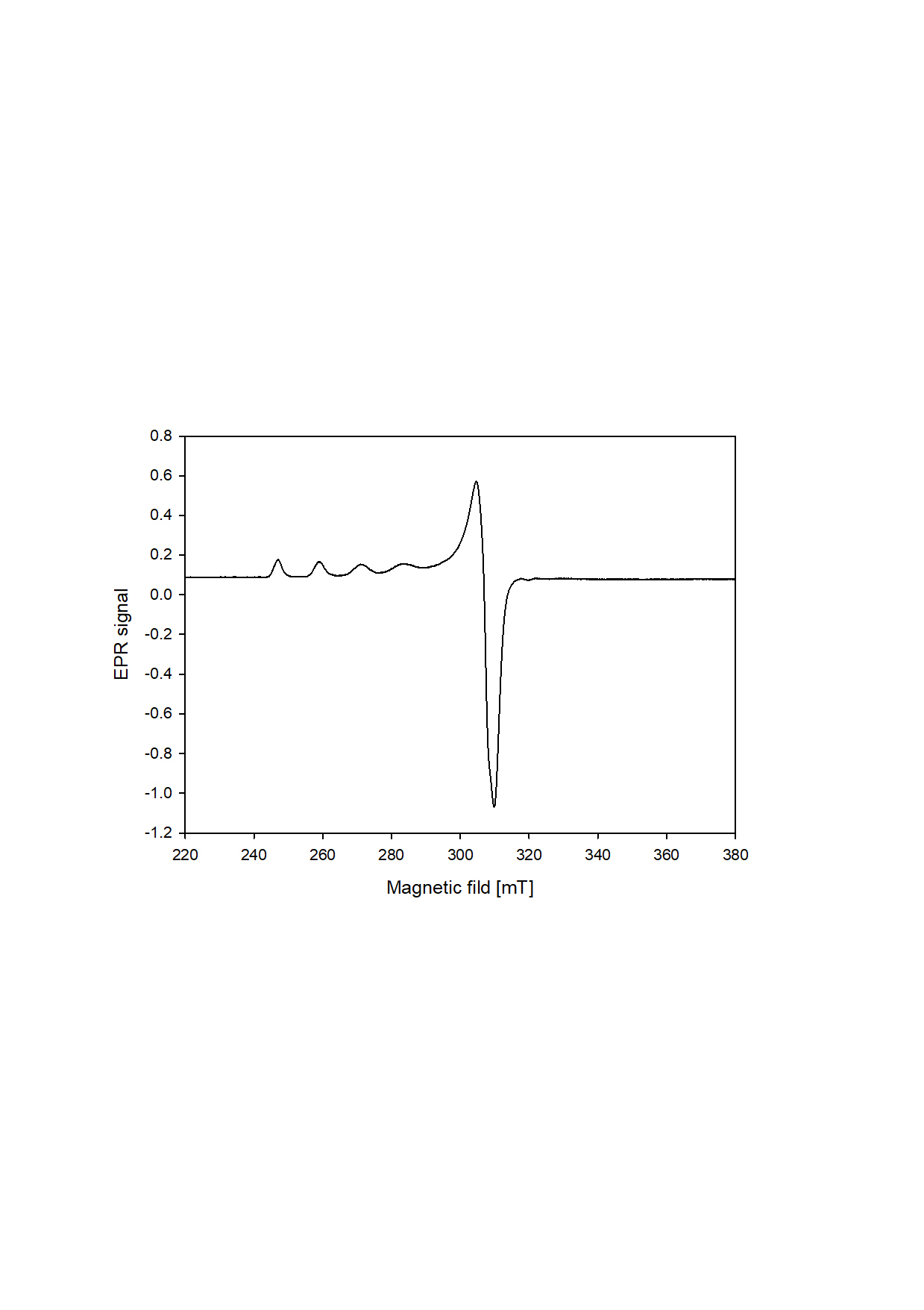
**

Figure S1. EPR spectrum of Cu(cholP)H_3_(CMP)complex at pH=2.5


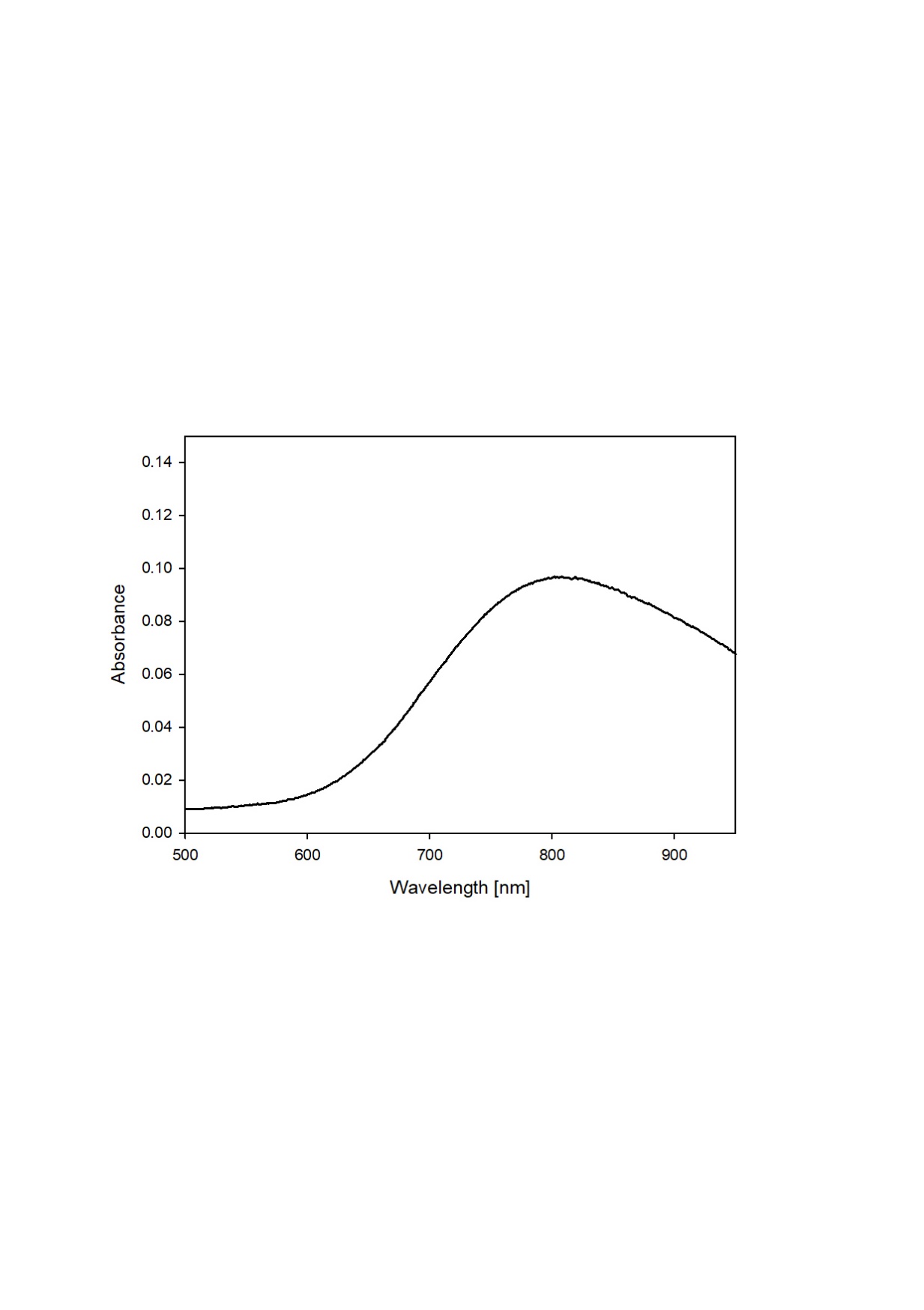


Figure S2. UV-Vis spectrum Cu(cholP)H_3_(CMP)complex at pH=2.5


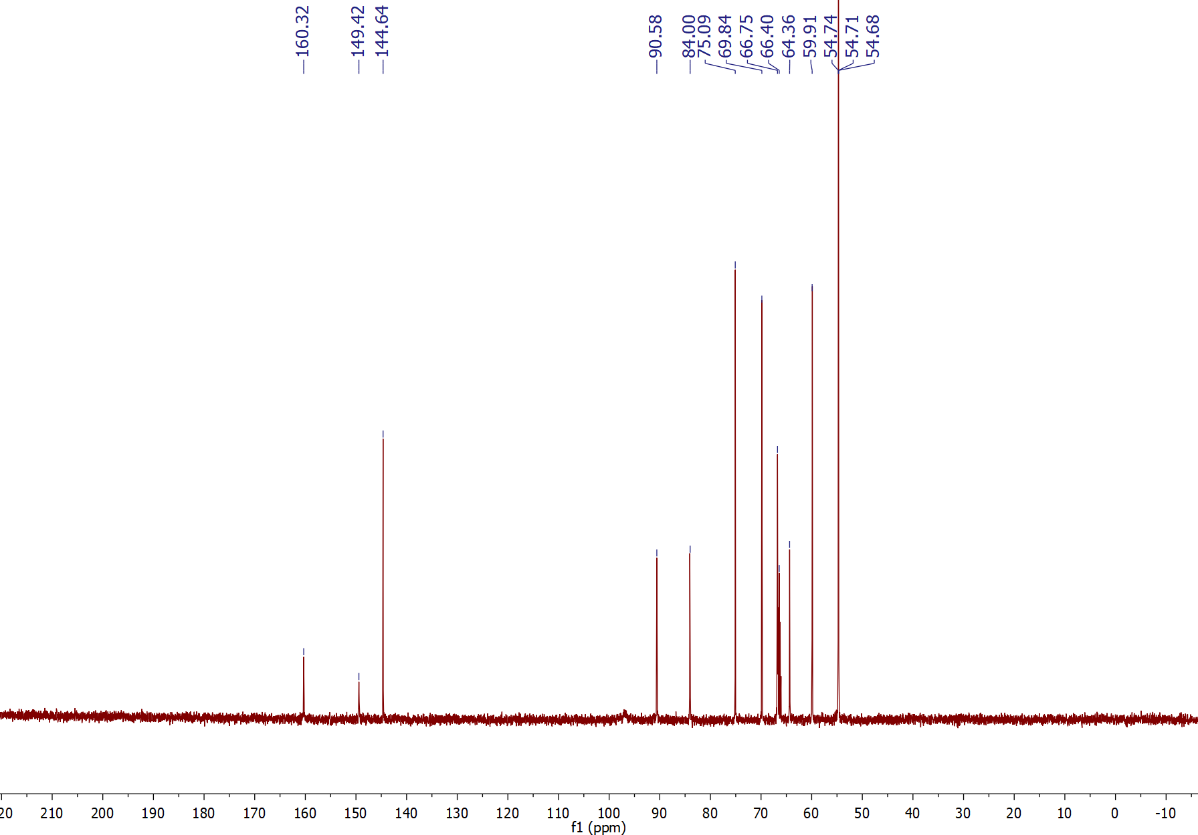


Figure S3. Cu(cholP)H_3_(CMP) ^13^ C NMR spectrum


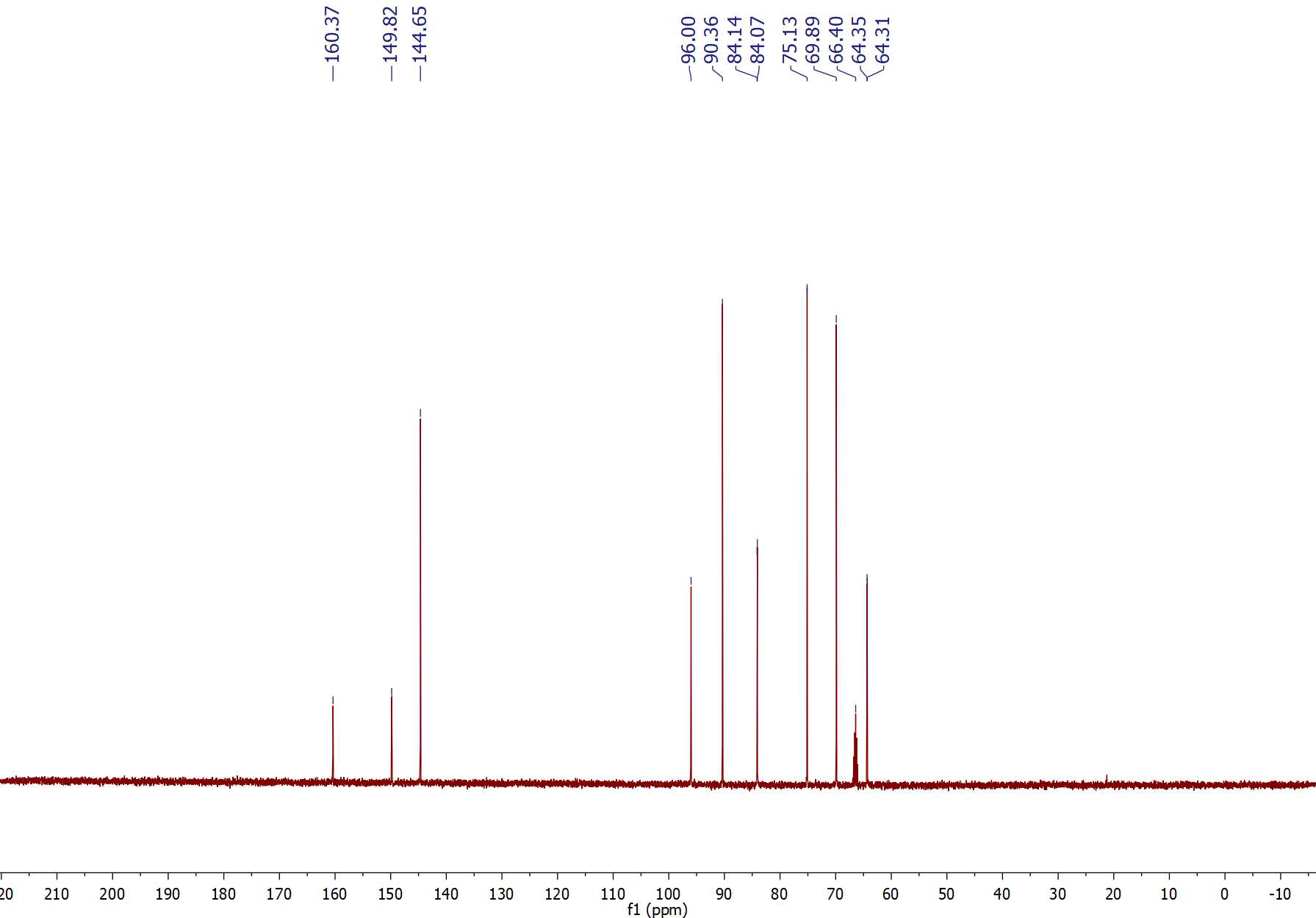


Figure S4. CMP ^13^ C NMR spectrum


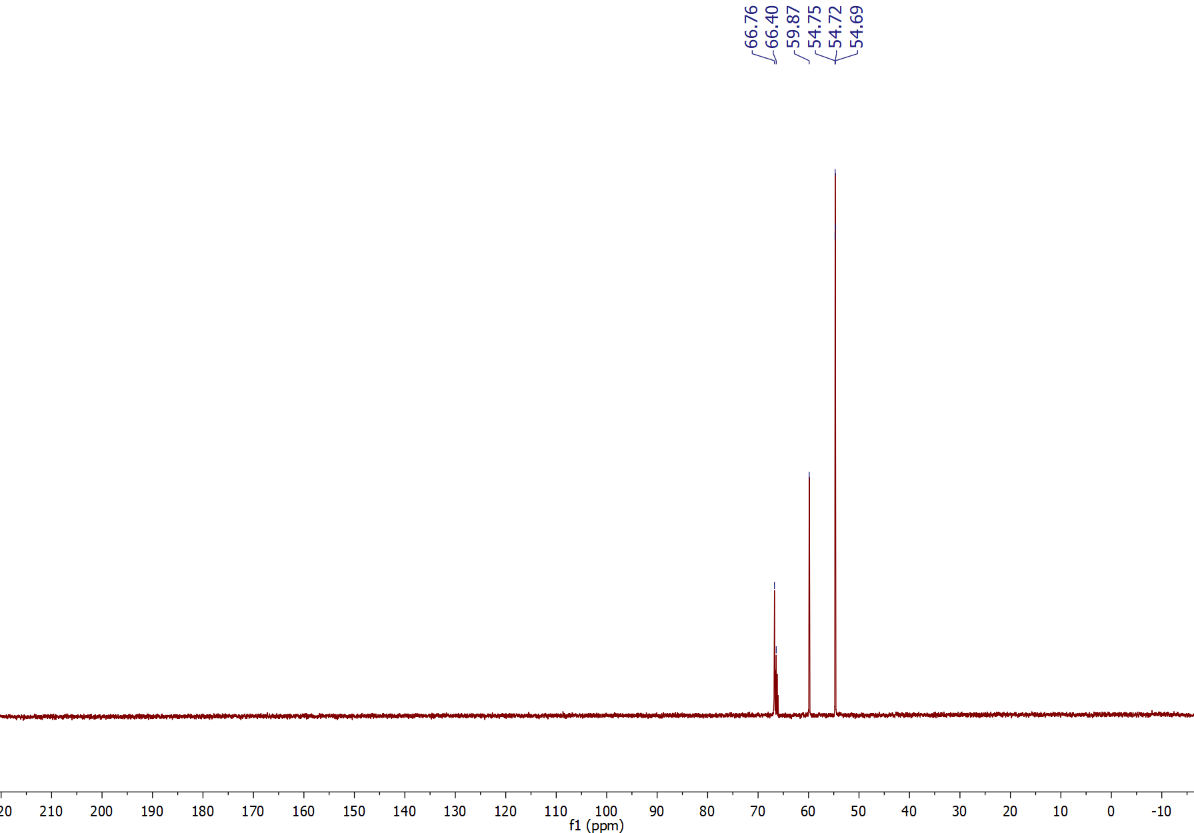


Figure S5. cholP ^13^ C NMR spectrum


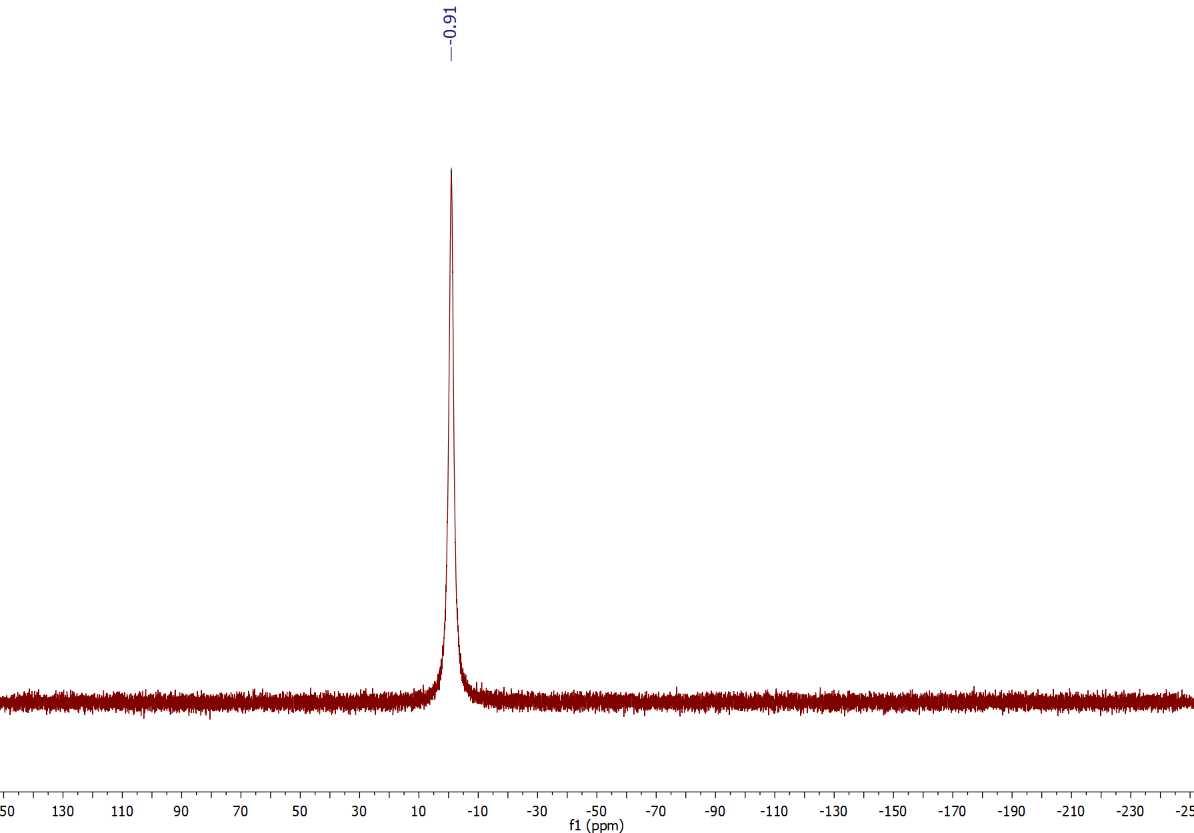


Figure S6. Cu(cholP)H_3_(CMP) ^31^ P NMR spectrum


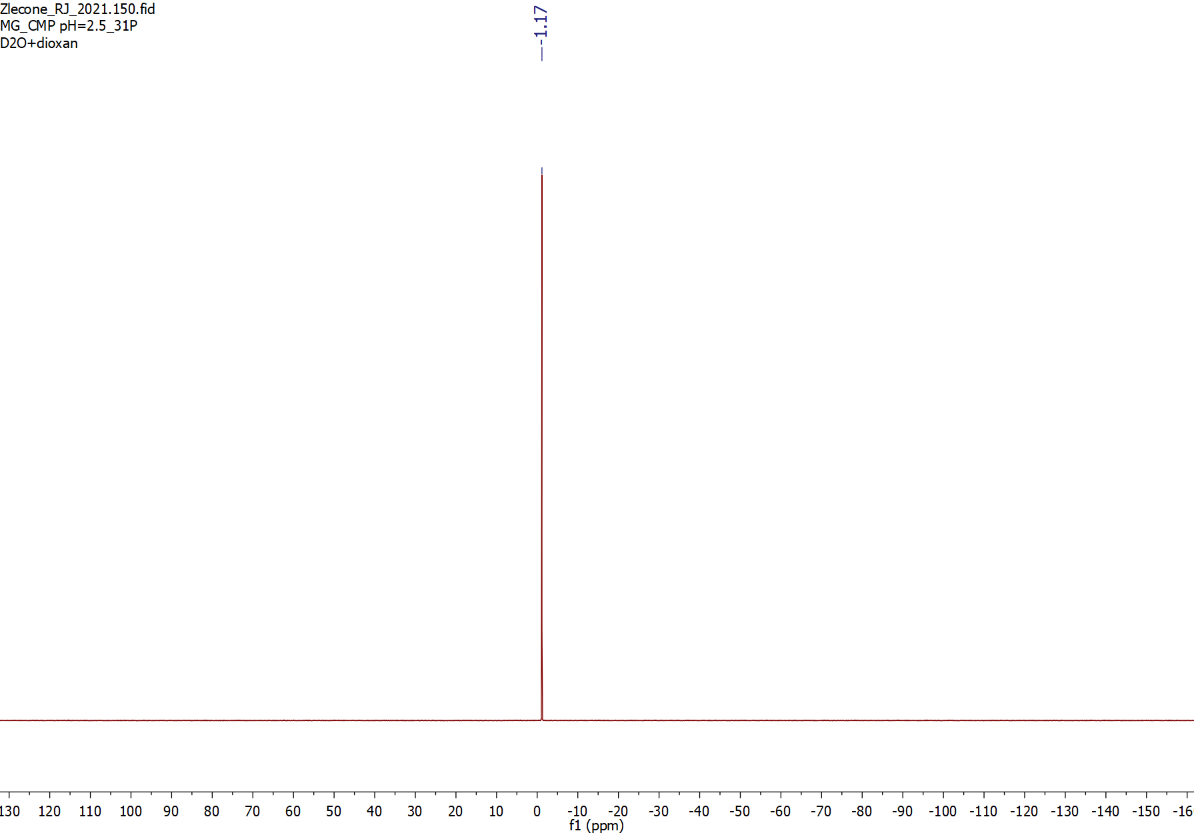


Figure S7. CMP ^31^ P NMR spectrum


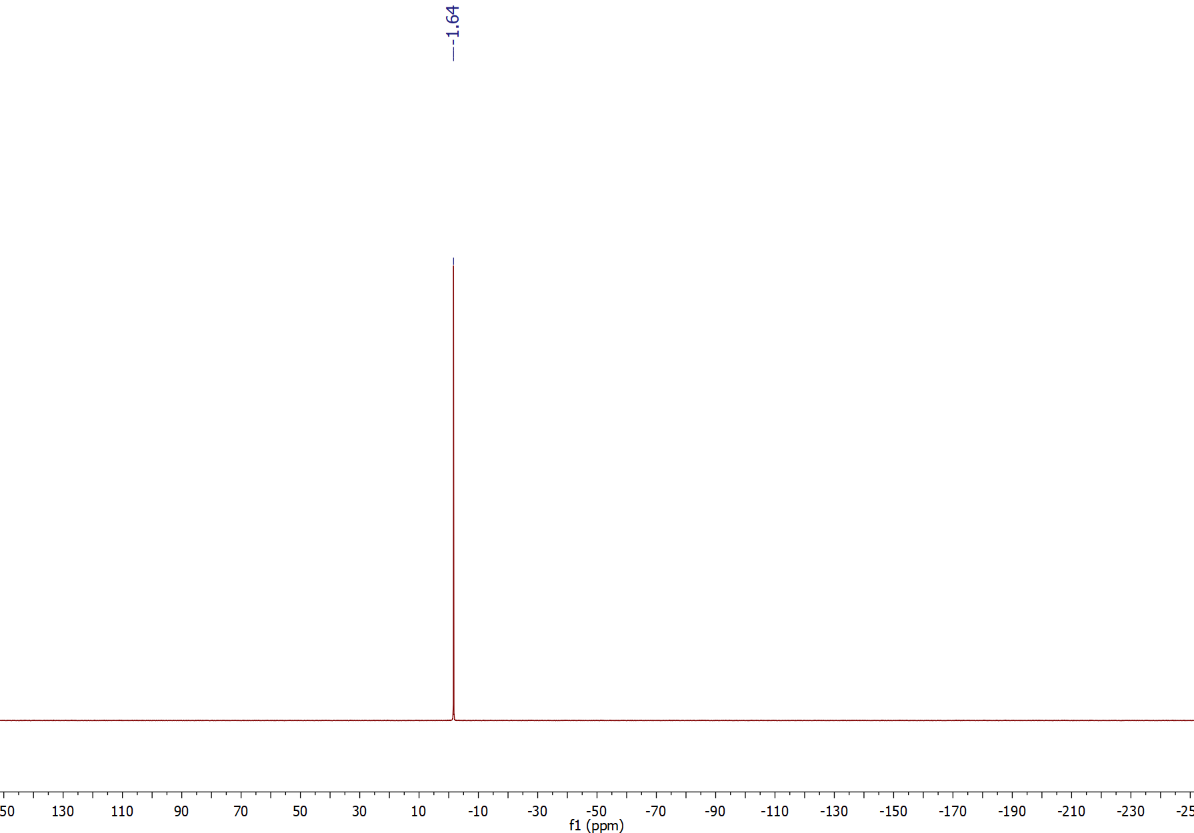


Figure S8. cholP ^31^ P NMR spectrum


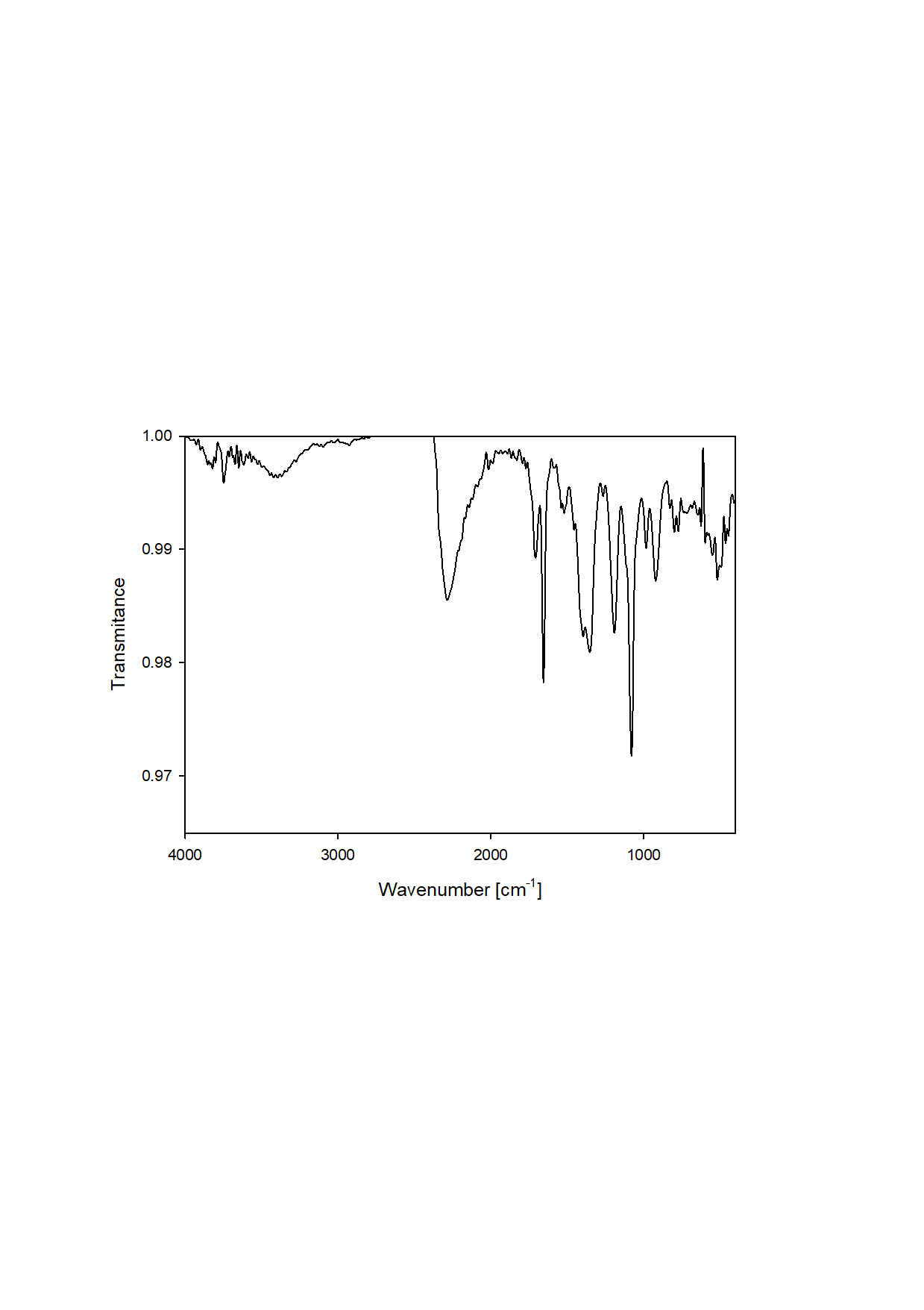


Figure S9. Cu(cholP)H_3_(CMP) FT-IR spectrum


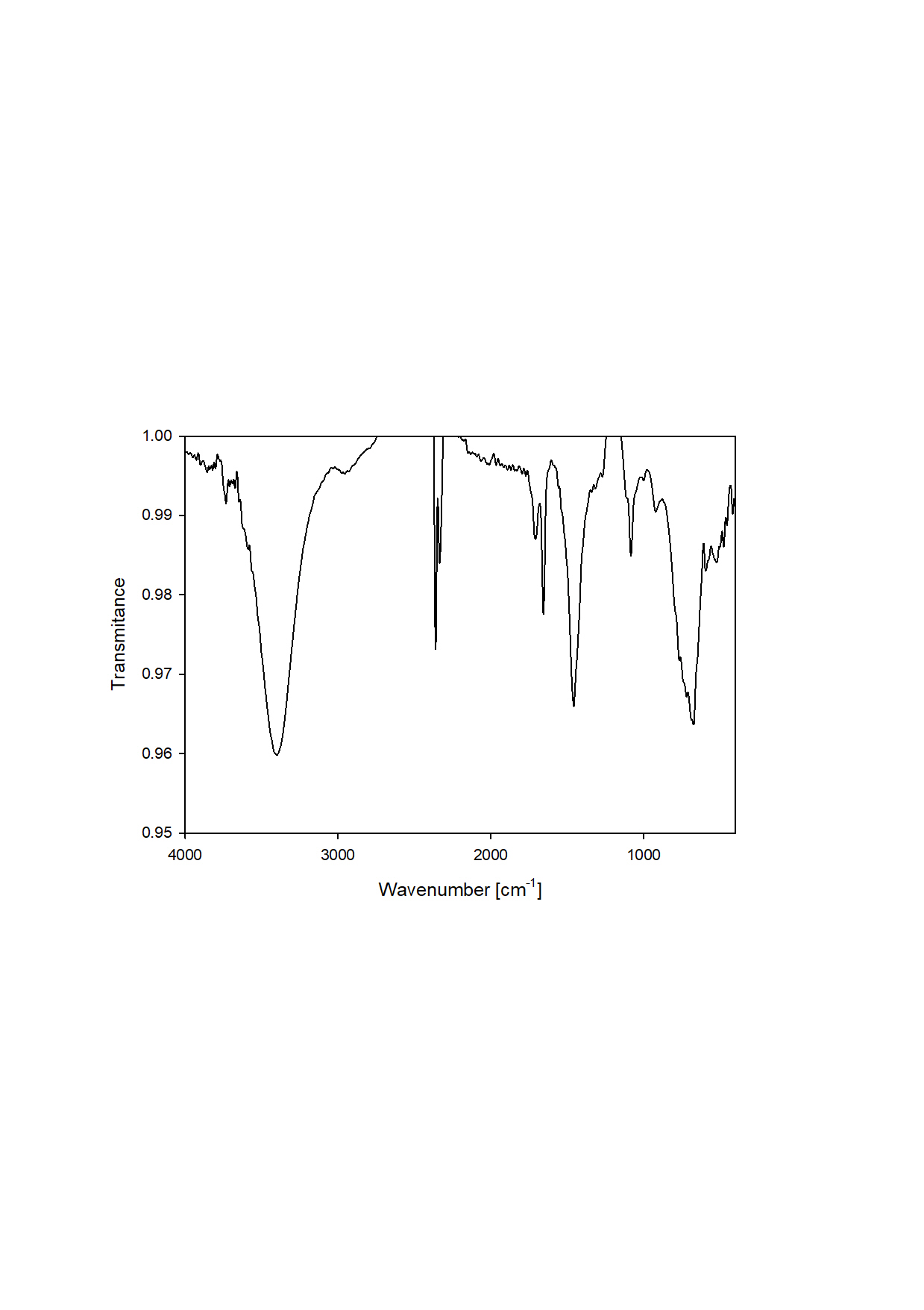


Figure S10. CMP FT-IR spectrum


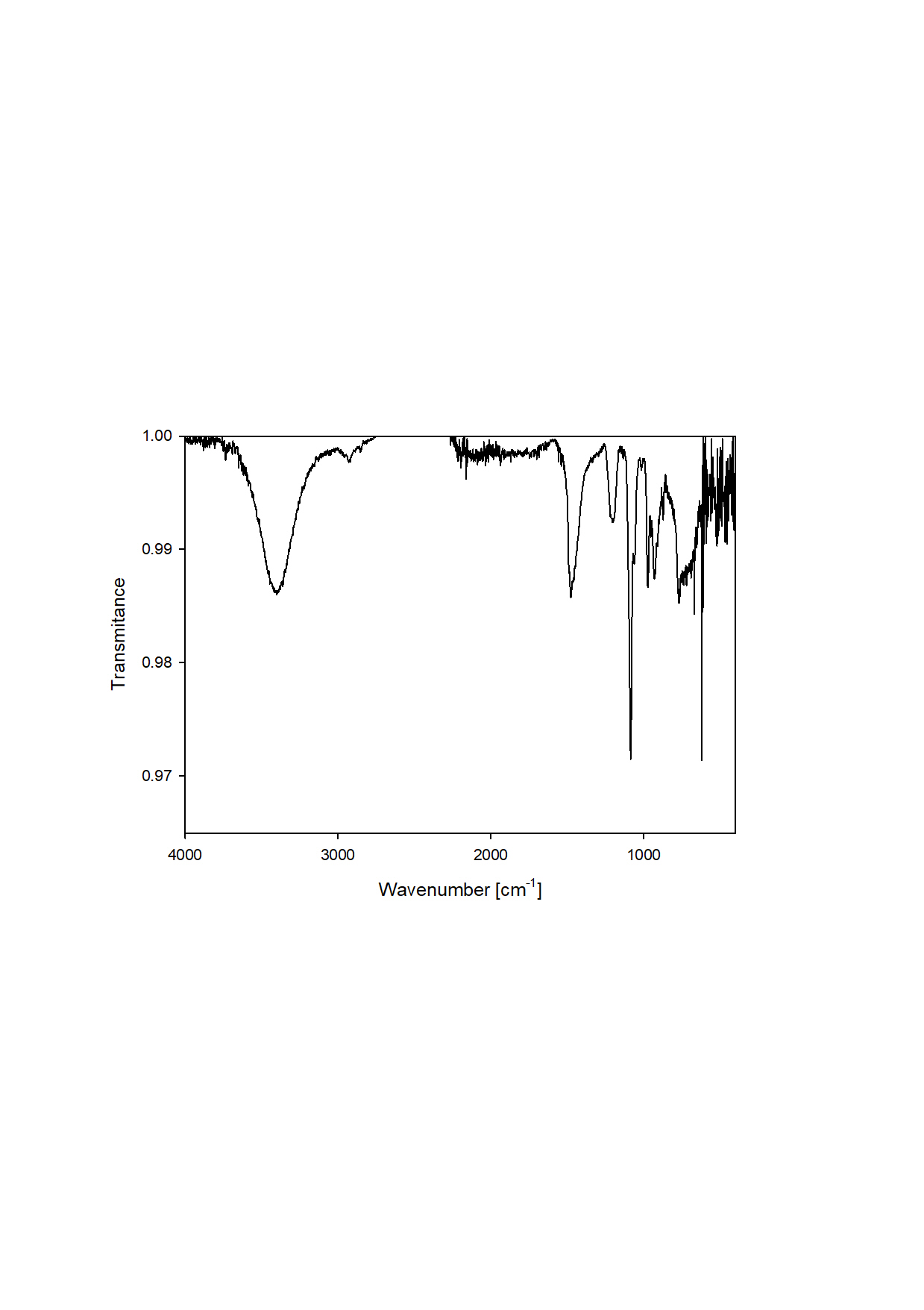


Figure S11. cholP FT-IR spectrum


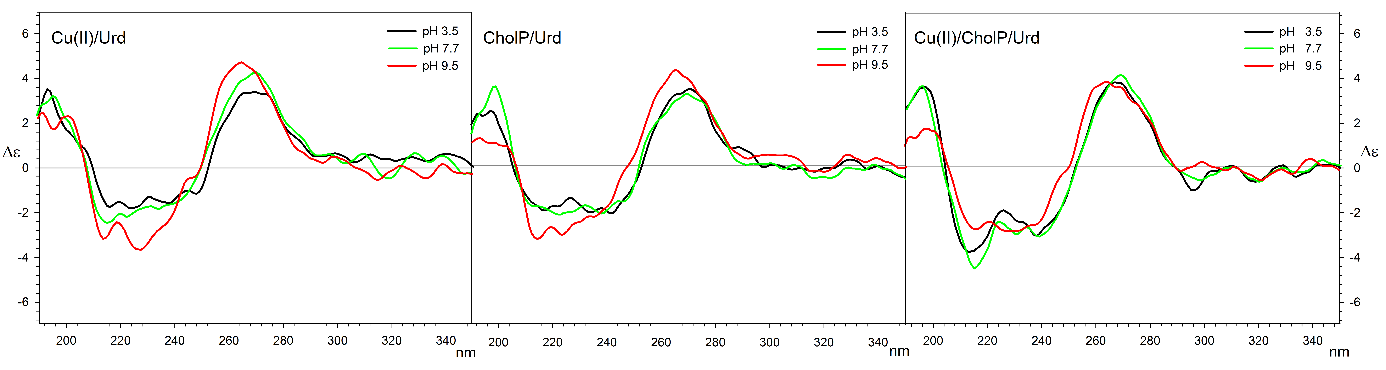


Figure S12. The set of CD spectra at different pH for Cu(II)/Urd, cholP/Urd and Cu(II)/cholP/Urd systems.

|  | Δε (nm) | | |
| --- | --- | --- | --- |
| Cu(II)/Urd | pH 3.5 | pH 7.7 | pH 9.5 |
|  | 3.39 (269) | 4.30 (271) | 4.72 (265) |
|  | -1.15 (248) |  |  |
|  | -1.58 (238) | -1.82 (235) |  |
|  | -1.79 (224) | -2.17 (223) | -3.66 (228) |
|  | -1.75 (216) | -2.43 (216) | -3.17 (214) |
|  | 3.53 (193) | 3.25 (196) | 2.31 (201) |
| CholP/Urd | pH 3.5 | pH 7.7 | pH 9.5 |
|  | 3.52 (271) | 3.33 (270) | 4.22 (265) |
|  | -2.02 (241) | -2.00 (238) | -2.35 (235) |
|  | -1.98 (234) |  | -3.17 (233) |
|  | -1.90 (217) | -2.04 (222) | -3.34 (214) |
|  | 2.55 (197) | 3.68 (199) | 1.16 (193) |
| Cu(II)/cholP/Urd | pH 3.5 | pH 7.7 | pH 9.5 |
|  | 3.82 (267) | 4.50 (269) | 3.85 (264) |
|  | -3.03 (238) | -3.07 (239) |  |
|  | -3.76 (213) | -2.96 (230) | -2.82 (228) |
|  | 3.64 (197) | -4.50 (215) | -2.73 (216) |
|  |  | 3.64 (196) | 1.73 (197) |

Table S1. Cotton effects maxima for Cu(II)/Urd, cholP/Urd and Cu(II)/cholP/Urd complexes in water solutions at C=2*10^-4^ mol*dm^-1^.


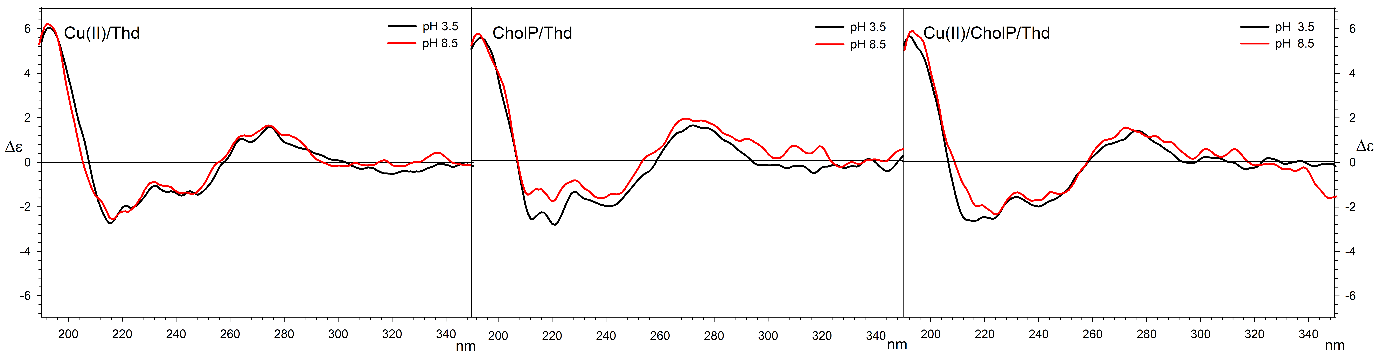


Figure S13. The set of CD spectra at different pH for Cu(II)/Thd, cholP/Thd and Cu(II)/cholP/Thd systems.

|  | Δε (nm) | |
| --- | --- | --- |
| Cu(II)/Thd | pH 3.5 | pH 8.5 |
|  | 1.60 (275) | 1.68 (275) |
|  | 1.07 (264) | 1.22 (265) |
|  | -1.46 (248) | -1.39 (247) |
|  | -1.49 (242) |  |
|  | -2.02 (224) |  |
|  | -2.72 (216) | -2.53 (217) |
|  | 6.05 (193) | 6.26 (193) |
| CholP/Thd | pH 3.5 | pH 8.5 |
|  | 1.66 (272) | 2.10 (270) |
|  | -1.97 (241) | -1.58 (238) |
|  | -2.81 (221) | -1.71 (220) |
|  | -2.54 (213) | -1.42 (211) |
|  | 5.60 (194) | 5.82 (193) |
| Cu(II)/cholP/Thd | pH 3.5 | pH 8.5 |
|  | 1.42 (277) | 1.56 (272) |
|  |  | -1.41 (248) |
|  | -1.98 (240) | -1.73 (237) |
|  | -2.55 (223) | -2.35 (224) |
|  | -2.63 (216) | -1.97 (217) |
|  | 5.69 (192) | 5.91 (193) |

Table S2. Cotton effects maxima for Cu(II)/Thd, cholP/Thd and Cu(II)/cholP/Thd complexes in water solutions at C=2*10^-4^ mol*dm^-1^.

**
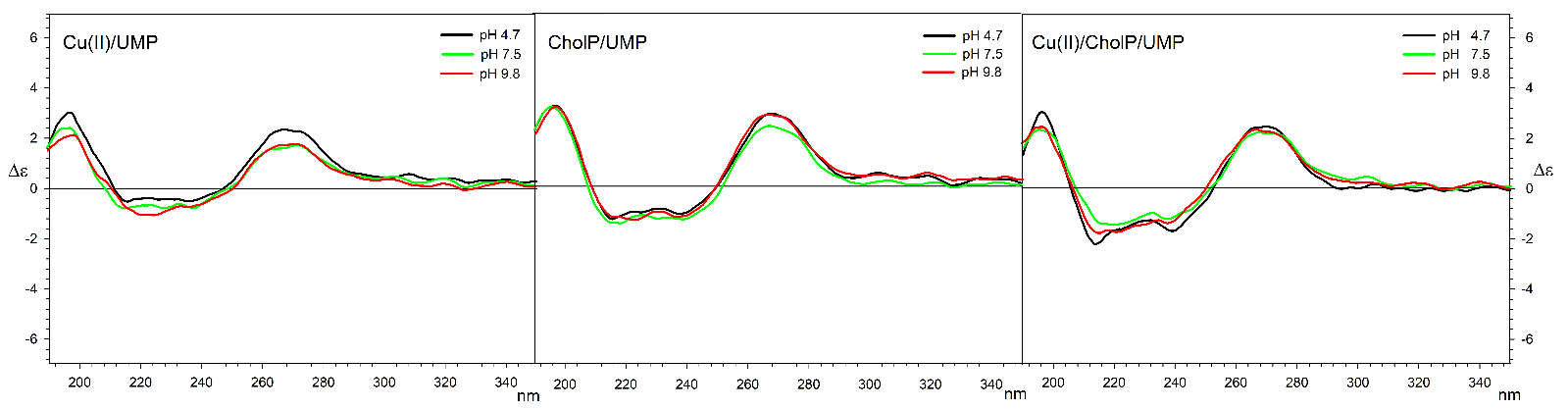
**

Figure S14. The set of CD spectra at different pH for Cu(II)/UMP, cholP/UMP and Cu(II)/cholP/UMP systems.

|  | Δε (nm) | | |
| --- | --- | --- | --- |
| Cu(II)/UMP | pH 4.7 | pH 7.5 | pH 9.8 |
|  | 2.35 (267) | 1.74 (272) | 1.76 (272) |
|  | -0.51 (236) | -0.75 (238) |  |
|  |  | -0.74 (228) | -1.06 (225) |
|  | -0.52 (215) | -0.76 (215) |  |
|  | 3.02 (197) | 2.44 (198) | 2.13 (198) |
| CholP/UMP | pH 4.7 | pH 7.5 | pH 9.8 |
|  | 2.99 (268) | 2.53 (267) | 2.81 (267) |
|  | -1.00 (237) | -1.19 (239) | -1.24 (236) |
|  |  | -1.15 (231) | -1.37 (223) |
|  | -1.22 (215) | -1.35 (218) | -1.20 (215) |
|  | 3.29 (197) | 3.28 (196) | 3.13 (197) |
| Cu(II)/CholP/UMP | pH 4.7 | pH 7.5 | pH 9.8 |
|  | 2.47 (270) | 2.23 (267) | 2.35 (267) |
|  | -1.68 (239) | -1.21 (237) | -1.38 (238) |
|  | -2.21 (214) | -1.45 (220) | -1.77 (215) |
|  | 3.05 (196) | 2.34 (195) | 2.45 (196) |

Table S3. Cotton effects maxima for Cu(II)/UMP, cholP/ UMP and Cu(II)/cholP/ UMP complexes in water solutions at C=2*10^-4^ mol*dm^-1^.

**
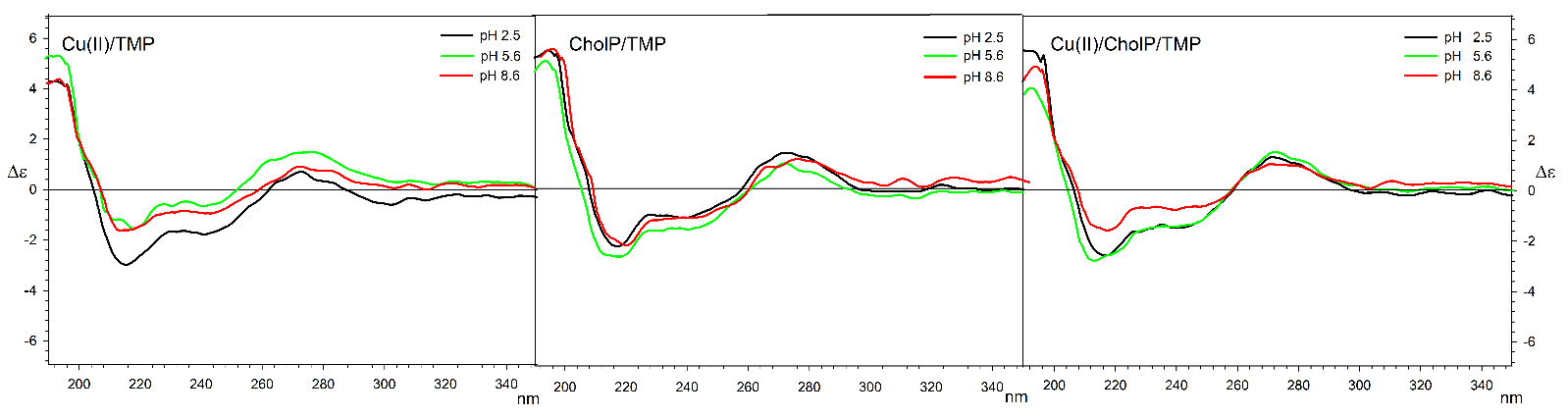
**

Figure S15. The set of CD spectra at different pH for Cu(II)/TMP, cholP/TMP and Cu(II)/cholP/TMP systems.

|  | Δε (nm) | | |
| --- | --- | --- | --- |
| Cu(II)/TMP | pH 2.5 | pH 5.6 | pH 8.6 |
|  | 0.70 (272) | 1.57 (277) | 0.96 (274) |
|  | -1.77 (241) | -0.60 (242) | -0.90 (242) |
|  | -2.99 (216) | -1.47 (219) | -1.58 (214) |
|  | 4.28 (192) | 5.38 (194) | 4.43 (194) |
| CholP/TMP | pH 2.5 | pH 5.6 | pH 8.6 |
|  | 1.50 (274) | 1.12 (272) | 0.89 (274) |
|  | -1.09 (240) | -1.50 (240) | -1.45 (239) |
|  | -2.20 (217) | -2.57 (217) | -2.54 (218) |
|  | 5.56 (195) | 5.18 (194) | 5.25 (194) |
| Cu(II)/CholP/TMP | pH 2.5 | pH 5.6 | pH 8.6 |
|  | 1.33 (271) | 1.52 (272) | 1.05 (271) |
|  | -1.46 (240) | -1.43 (239) | -0.77 (240) |
|  | -2.57 (217) | -2.79 (213) | -1.57 (218) |
|  |  | 4.07 (192) | 4.92 (194) |

Table S4. Cotton effects maxima for Cu(II)/TMP, cholP/ TMP and Cu(II)/cholP/ TMP complexes in water solutions at C=2*10^-4^ mol*dm^-1^.
